# The Architecture of Selectivity: Molecular Basis of BlTx2 Discrimination Among Kv Channels and Kv4 Isoforms

**DOI:** 10.3390/toxins18100431

**Published:** 2026-10-08

**Authors:** Yuval Levy, Ayalon Reis, Galit Blecher, Oksana Daron, Sara Dadon, Raz Zarivach, Noam Zilberberg

**Affiliations:** 1 Department of Life Sciences, Ben-Gurion University of the Negev, P.O. Box 653, Beer-Sheva 8410501, Israel; levyyu@post.bgu.ac.il (Y.L.); ayalon16@gmail.com (A.R.); galit.bleicher@nki.rfmh.org (G.B.); daron.oksana@gmail.com (O.D.); sarahd@bgu.ac.il (S.D.); zarivach@bgu.ac.il (R.Z.); 2 The Zelman Center for Brain Science Research, Ben-Gurion University of the Negev, P.O. Box 653, Beer-Sheva 8410501, Israel

**Keywords:** scorpion toxin, α-KTx15, voltage-gated potassium channel, Kv4 potassium channels, toxin selectivity, A-type potassium current

## Abstract

Kv4 channels generate transient A-type potassium currents, but the contribution of Kv4.1 remains difficult to define because few pharmacological tools discriminate among Kv4 isoforms. We investigated the molecular basis of Kv4.1-selective inhibition by BlTx2, an α-KTx15 scorpion toxin from *Buthacus leptochelys*. Using electrophysiology, mutagenesis, tandem-channel analysis, structural modeling, and evolutionary analysis, we mapped the determinants governing BlTx2 activity and selectivity. Substitution of conserved α-KTx residues Lys27 and Tyr36 abolished detectable inhibition, whereas N30A reduced potency, supporting a canonical pore-blocking mechanism. A Kv4.1/Kv4.3 tandem chimera reduced BlTx2 potency, consistent with a contribution of adjacent Kv4.1 subunits to the high-affinity toxin-binding surface. Channel mutagenesis identified the Kv4.2/Kv4.3 lysine corresponding to Kv4.1 Asn355 as a key barrier to BlTx2 activity, since introducing this lysine into Kv4.1 abolished detectable inhibition. Mutational mapping of BlTx2 revealed that pore-facing residues required for inhibition are separated from peripheral residues that tune potency and cross-reactivity. In particular, Q9K and I18K conferred Kv1.2 activity, partly through interaction with Kv1.2 Glu355. Evolutionary analysis linked conserved pore-facing residues to purifying selection, and peripheral determinants to diversification. These findings identify distinct conserved and peripheral determinants that together shape BlTx2 activity and selectivity, thereby establishing BlTx2 as a useful probe for Kv4.1 function.

## 1. Introduction

Voltage-gated potassium channels of the Kv4 subfamily, Kv4.1, Kv4.2, and Kv4.3, mediate transient A-type K^+^ currents that play central roles in the control of membrane excitability, action potential waveform, firing frequency, and dendritic signal integration in excitable cells [1,2]. Although these three channels share closely related core biophysical properties, they differ in tissue distribution and physiological contribution [1,2]. Their functional properties are further shaped by auxiliary subunits such as KChIPs and DPP-like proteins, complicating the assignment of isoform-specific functions in native systems [3,4].

Beyond their physiological roles, Kv4 channels are clinically relevant because altered A-type current can disturb neuronal and cardiac excitability [1,2]. Human variants in KCND1/Kv4.1 and KCND2/Kv4.2 have been associated with neurodevelopmental disorders, including epilepsy [5,6], and a KCND2/Kv4.2 mutation has also been linked to nocturnal paroxysmal atrial fibrillation [7]. KCND3/Kv4.3 variants have been linked to spinocerebellar ataxia 19/22 and cardiac arrhythmia syndromes [8,9,10]. Because Kv4 channels have overlapping biophysical properties but distinct physiological and disease associations, isoform-selective ligands are particularly valuable for dissecting the contribution of individual Kv4 channels without the interpretive limitations imposed by broad-spectrum blockers or genetic manipulation.

Venom-derived peptides have provided some of the most informative probes for potassium-channel structure and function because they bind with high affinity to defined extracellular receptor surfaces and can report subtle differences in channel architecture [11,12,13]. Among peptides that affect Kv4 channels, two broad mechanistic classes have been described [14,15]. Kv4-active spider peptides generally act as gating modifiers by targeting the voltage-sensor domain. In contrast, short scorpion α-KTx peptides act as pore blockers by binding at the external vestibule of the channel [14,15]. This distinction is important because pore-blocking α-KTx peptides are especially amenable to residue-level structure-function analysis: their activity reflects the geometry, electrostatics, and ion occupancy of the outer pore rather than voltage-sensor movement [15,16].

α-KTx scorpion toxins constitute a well-established class of peptide inhibitors that target voltage-gated potassium (Kv) channels by binding to the external vestibule of the pore and occluding ion conduction. The conceptual framework for this interaction emerged from early mutagenesis studies demonstrating that residues within the pore-forming S5-S6 linker of Shaker channels determine sensitivity to charybdotoxin (CTX), thereby localizing the toxin receptor to the outer mouth of the pore [17,18]. Subsequent kinetic and functional analyses established that a single toxin molecule blocks a single channel and that a conserved lysine residue, typified by Lys27 in CTX, protrudes into the conduction pathway to occlude K^+^ flux physically [18].

Systematic mapping of both toxin and channel surfaces further refined this model. Mutagenesis studies of CTX and Shaker identified complementary interaction surfaces, demonstrating that toxin binding is mediated by a defined set of residues at the channel vestibule and the toxin surface [19], while independent analyses of toxin variants established the functional topography of the toxin binding interface [20]. A key advance was the demonstration that transferring the pore region between Kv channel subtypes transfers toxin sensitivity, providing direct evidence that the binding site resides within the outer vestibule of the pore [21]. A related structural principle is the functional dyad, typically composed of the pore-inserting lysine and a nearby aromatic or hydrophobic residue, often positioned approximately 6–7 Å apart, that contributes to toxin orientation and channel recognition [15,22,23,24].

High-resolution functional analyses using α-KTx toxins such as agitoxin-2 (AgTx2) established the structural basis of toxin-channel recognition. Double-mutant cycle analysis revealed energetically coupled residue pairs between the toxin and the channel, thereby allowing inference of the toxin’s relative position at the pore entrance [25]. Parallel structural work provided NMR-derived toxin structures, enabling integration of functional data into three-dimensional models of the toxin-channel complex [26]. These approaches were extended to multiple toxins and channels, establishing a generalizable framework in which α-KTx peptides act as “molecular calipers” to probe the geometry of the outer vestibule and selectivity filter region [27].

Further refinement of this model identified specific contact pairs and positioned the toxin relative to the selectivity filter. Mutant-cycle analysis identified energetically coupled toxin-channel residue pairs [28], while complementary studies localized the selectivity filter signature sequence (GYGD motif) within the toxin-binding interface [29]. Additional biochemical and functional mapping defined the footprint of toxins such as AgTx2 on the pore surface [30] and demonstrated that the conserved lysine side chain aligns with the selectivity filter axis, functionally coupling toxin binding to ion permeation [31].

Direct structural validation of this pore-blocking mechanism was provided by the crystal structure of a CTX-Kv channel complex, which showed the toxin positioned in the external vestibule, with its conserved lysine side chain inserted into the pore, physically occluding the ion-conduction pathway [15]. Collectively, these studies establish a coherent mechanistic model in which α-KTx scorpion toxins act as high-affinity pore blockers that bind to the extracellular vestibule of Kv channels and inhibit conduction by directly occluding the selectivity filter region.

Many α-KTx peptides contain what has often been described as a functional dyad, typically a pore-inserting lysine paired with a nearby aromatic or hydrophobic residue that contributes to channel recognition [15,23,24]. However, later mutational work showed that this concept is useful but not absolute [23,24]. Thus, for α-KTx peptides, conserved lysine-centered pore block represents only part of the mechanism, whereas subtype selectivity is often encoded by additional residues that tune complementary contacts with the outer vestibule [15,23,24].

Several α-KTx15 peptides, the principal scorpion pore blockers known to affect Kv4-mediated currents [4,32,33], including Aa1, BmTX3, AaTx1, and Discrepin, have been reported to inhibit A-type currents in native neuronal preparations [33,34,35,36]. AmmTX3 was likewise shown to block Kv4-mediated A-type currents and to act on Kv4 channels in heterologous systems [4,32]. Together, these findings support the view that α-KTx15 peptides are major scorpion pore blockers associated with Kv4-mediated currents [4,32,33]. Mutational analyses indicate that both conserved and toxin-specific surface determinants govern Kv-channel recognition by α-KTx15 peptides. In BmTX3, deletion of the C-terminal Tyr36-Pro37 residues abolished inhibition of A-type K^+^ currents while preserving activity on hERG, suggesting that the C-terminal face containing the classical lysine-aromatic dyad contributes selectively to Kv4-like channel block. In contrast, hERG recognition involves a distinct interaction surface. Consistent with this idea, studies of AmmTX3 identified Arg18 and Lys19 as critical residues for hERG inhibition, since alanine substitution of either residue markedly reduced hERG block, defining an α-helical basic “hot spot” that is functionally separable from the canonical pore-plugging lysine [37]. Mutational studies of Discrepin further support the importance of electrostatic surface complementarity in α-KTx15 toxins. Introduction of positive charges, particularly V6K and D20K, enhanced the block of native neuronal A-type currents, and the V6K-D20K double mutant increased apparent affinity for Kv4.3 by approximately tenfold, whereas removal or neutralization of Lys13 severely impaired activity, reducing apparent affinity by more than twentyfold [38,39].

Previous work from our laboratory identified two members of the α-KTx15 subfamily from *Buthacus leptochelys*, BlTx1 and BlTx2, and showed that both inhibit Kv4.1 while sparing Kv4.2 and Kv4.3 [40]. Among the channels examined, BlTx2 behaved as a Kv4.1-selective blocker, whereas BlTx1 retained an additional activity against Kv1.2 [40]. Because the two peptides differ by only 5 residues, they form an unusually informative pair for defining how minimal sequence divergence can preserve strong Kv4.1 inhibition while altering cross-reactivity with another Kv channel subtype [40].

Although α-KTx15 toxins constitute the principal group of scorpion pore blockers associated with Kv4-mediated A-type currents, the molecular basis of their activity and subtype selectivity remains only partially understood. Most studies on α-KTx15 toxins have focused on wild-type toxins, native A-type currents, or limited channel panels, leaving unresolved which toxin residues determine activity against Kv4 channels and which residues tune selectivity among closely related Kv subtypes. Previous findings suggest that a broader three-dimensional distribution of charged, hydrophobic, and steric determinants governs α-KTx15 activity and selectivity. Still, they do not define how individual residues contribute to discrimination among Kv4.1, Kv4.2, Kv4.3, and other Kv channels. Thus, despite the recognized pharmacological relevance of this subfamily, residue-level determinants of Kv4 isoform selectivity remain poorly defined. In the present study, we address this gap by using BlTx1 and BlTx2, closely related α-KTx15 peptides [40], and by applying a mutagenesis-based strategy to distinguish residues required for Kv4.1 activity from those responsible for subtype selectivity and cross-reactivity with other Kv channels.

## 2. Results

### 2.1. BlTx2 Interacts with Kv4.1 Through a Canonical α-KTx Pore-Blocking Mechanism

#### 2.1.1. Conserved α-KTx Functional Residues Contribute to BlTx2-Mediated Kv4.1 Block

**Figure 1:**
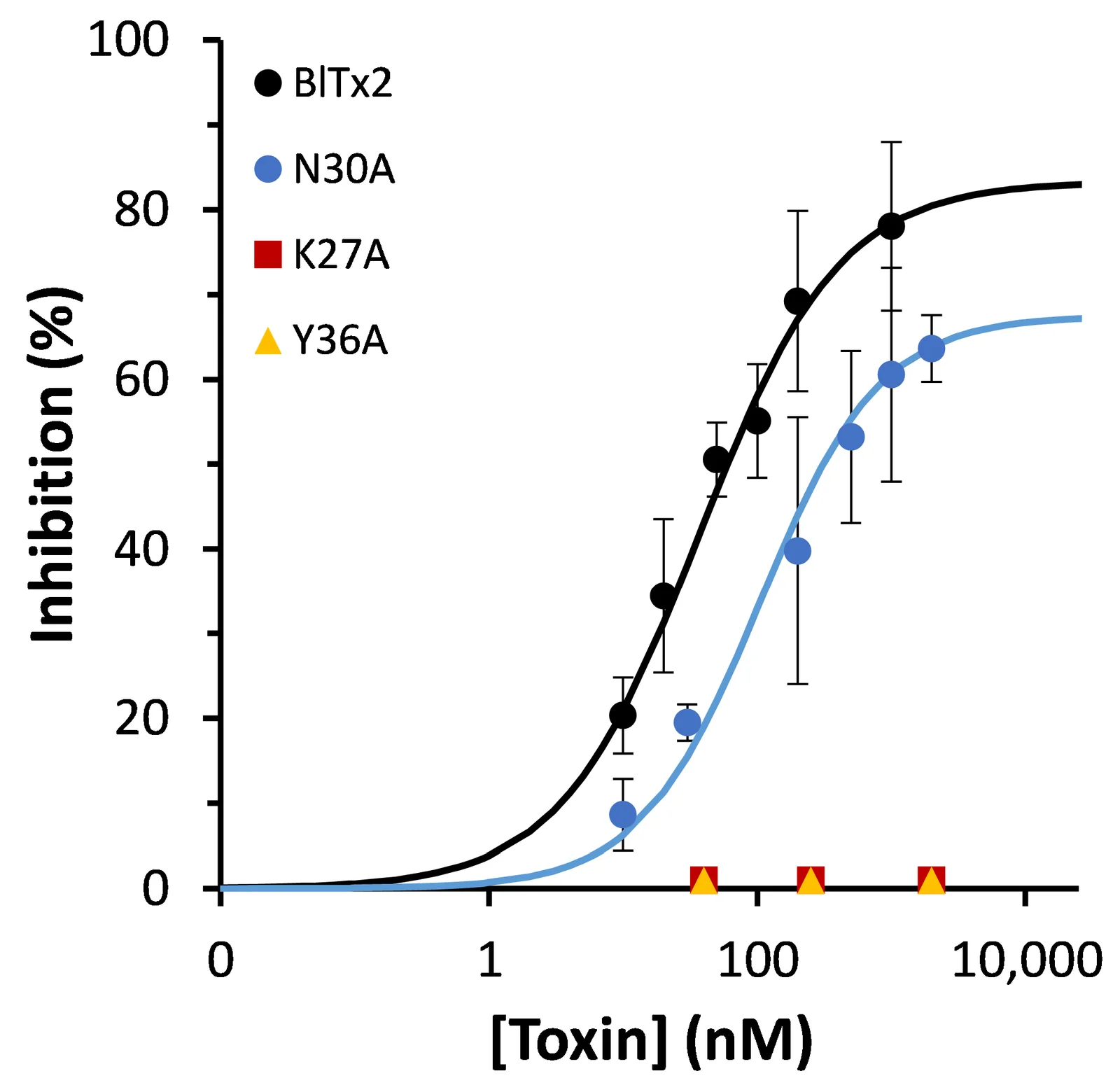
Concentration-dependent inhibition of Kv4.1 by BlTx2, and selected BlTx2 variants. Each point represents the mean ± S.E.M. (*n* = 3–5). Solid lines show fits of the data to a modified Hill equation: % inhibition = max/[1 + (IC_50_/[Tx])^h^], where max is the maximal inhibition, [Tx] is the toxin concentration in nM, IC_50_ is the toxin concentration producing 50% of maximal inhibition, and h is the Hill coefficient. WT BlTx2 (black) inhibited Kv4.1 with an IC_50_ of 35.6 ± 7.5 nM. BlTx2-N30A (blue) showed a significant reduction in potency, with an IC_50_ of 105 ± 20 nM. BlTx2-K27A (orange triangles) and BlTx2-Y36A (red squares) did not produce measurable inhibition at concentrations up to 2000 nM.

In our previous study [40], we identified two scorpion venom-derived α-KTx15 peptides, BlTx1 and BlTx2, and showed that both toxins display selectivity among Kv4 family isoforms, inhibiting Kv4.1 but not Kv4.2 or Kv4.3. Here, we used BlTx2 and a panel of BlTx2-derived variants to define the molecular determinants underlying its activity and selectivity. Because we previously found that the potency of WT BlTx2 was not significantly affected by the presence of KChIP2, with indistinguishable IC_50_ values obtained for Kv4.1 alone and the Kv4.1-KChIP2 construct [40], all BlTx2 variants in the present study were tested using the Kv4.1-KChIP2 chimera. First, we asked whether BlTx2, as a member of the α-KTx15 subfamily, blocks Kv channels through a mechanism similar to that described for other α-KTx subfamilies. Based on the established α-KTx pore-blocking mechanism, in which a conserved lysine protrudes into the channel pore and neighboring channel-facing residues, such as Asn30 and Tyr36, contribute to toxin-channel recognition [18,25,30], we examined the roles of the corresponding BlTx2 residues, Lys27, Asn30, and Tyr36, in Kv4.1 inhibition. We therefore tested the concentration-dependent inhibitory effects of three BlTx2 variants carrying substitutions at these positions. WT BlTx2 blocked Kv4.1 with an IC_50_ of 35.6 ± 7.5 nM, whereas BlTx2-N30A showed a significant reduction in potency, with an IC_50_ of 105 ± 20 nM (*p* = 0.0159). In contrast, BlTx2-K27A and BlTx2-Y36A produced no detectable inhibition at concentrations up to 2000 nM (Figure 1). These findings indicate that BlTx2 activity depends on conserved α-KTx functional positions, supporting the conclusion that BlTx2 blocks Kv4.1 through a mechanism related to the canonical α-KTx pore-blocking mode.

#### 2.1.2. BlTx2 Inhibition Is Consistent with a Multi-Subunit Binding Surface

**Figure 2:**
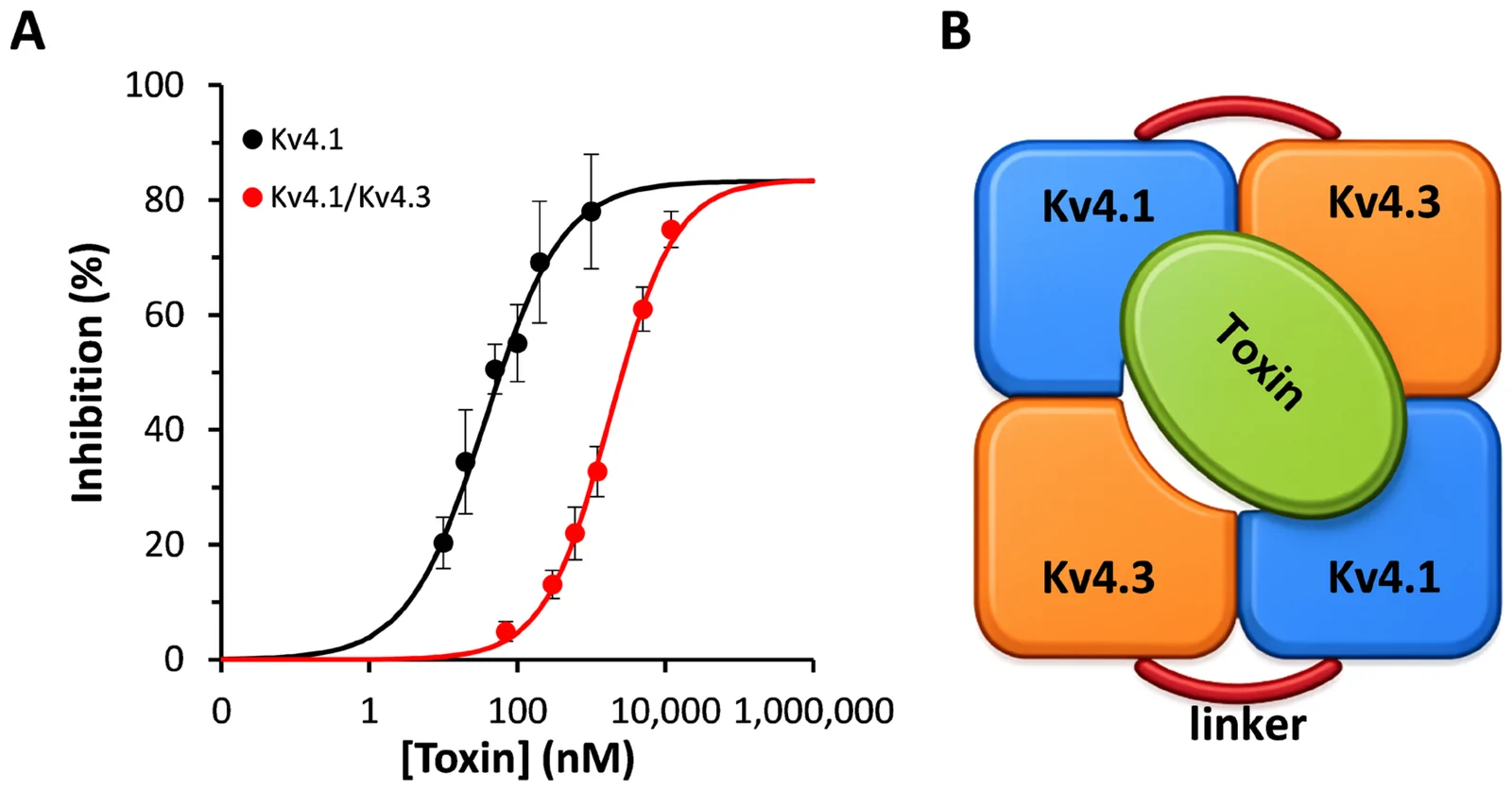
Reduced potency of BlTx2 on a Kv4.1/Kv4.3 tandem chimera. (**A**) Concentration-response curves showing inhibition of homotetrameric Kv4.1 and the Kv4.1/Kv4.3 tandem chimera by BlTx2. The tandem construct contained Kv4.1 and Kv4.3 subunits linked by a 10-Gln linker and was designed to generate channels with alternating Kv4.1 and Kv4.3 subunits. BlTx2 inhibited the chimera with an IC_50_ of 1767 ± 203 nM, compared with 35.6 ± 7.5 nM for homotetrameric Kv4.1. Data are shown as mean ± S.E.M. (*n* = 3–5), and solid lines represent fits to a modified Hill equation. (**B**) Schematic top view of BlTx2 (Toxin) bound to the Kv4.1/Kv4.3 tandem chimera, illustrating the predicted alternating arrangement of Kv4.1 and Kv4.3 subunits.

α-KTx toxins have been shown to interact with residues contributed by multiple channel subunits [15,18,25,30]. To test whether BlTx2 similarly requires a multi-subunit binding surface, we generated a Kv4.1/Kv4.3 tandem-dimer chimera. Each tandem dimer contained one Kv4.1 subunit linked to one Kv4.3 subunit by a 10-glutamine linker. Assembly of two such tandem dimers is expected to produce channels with alternating Kv4.1 and Kv4.3 subunits, placing identical subunits diagonally opposite one another and generating a bilaterally symmetric pore architecture. This expected arrangement is illustrated schematically in Figure 2B. Because BlTx2 does not detectably inhibit Kv4.3, reduced toxin potency for this alternating Kv4.1/Kv4.3 channel would be expected if high-affinity binding requires contributions from adjacent Kv4.1 subunits. Consistent with this prediction, BlTx2 inhibited the Kv4.1/Kv4.3 tandem-dimer chimera with markedly reduced potency compared with homotetrameric Kv4.1 channels (IC_50_ = 1767 ± 203 nM, *p* < 1 × 10^−3^; Figure 2A). Thus, the marked reduction in BlTx2 potency for the alternating Kv4.1/Kv4.3 channel is consistent with a contribution of adjacent Kv4.1 subunits to high-affinity toxin binding. However, because the tandem architecture itself could alter local pore geometry, subunit arrangement, or toxin accessibility, this experiment does not by itself establish the exact subunit contacts involved in BlTx2 binding.

### 2.2. Toxin and Channel Determinants Governing Kv4 Versus Kv1.2 Selectivity

#### 2.2.1. BlTx2 Residues Controlling Kv4.1/Kv1.2 Selectivity

**Figure 3:**
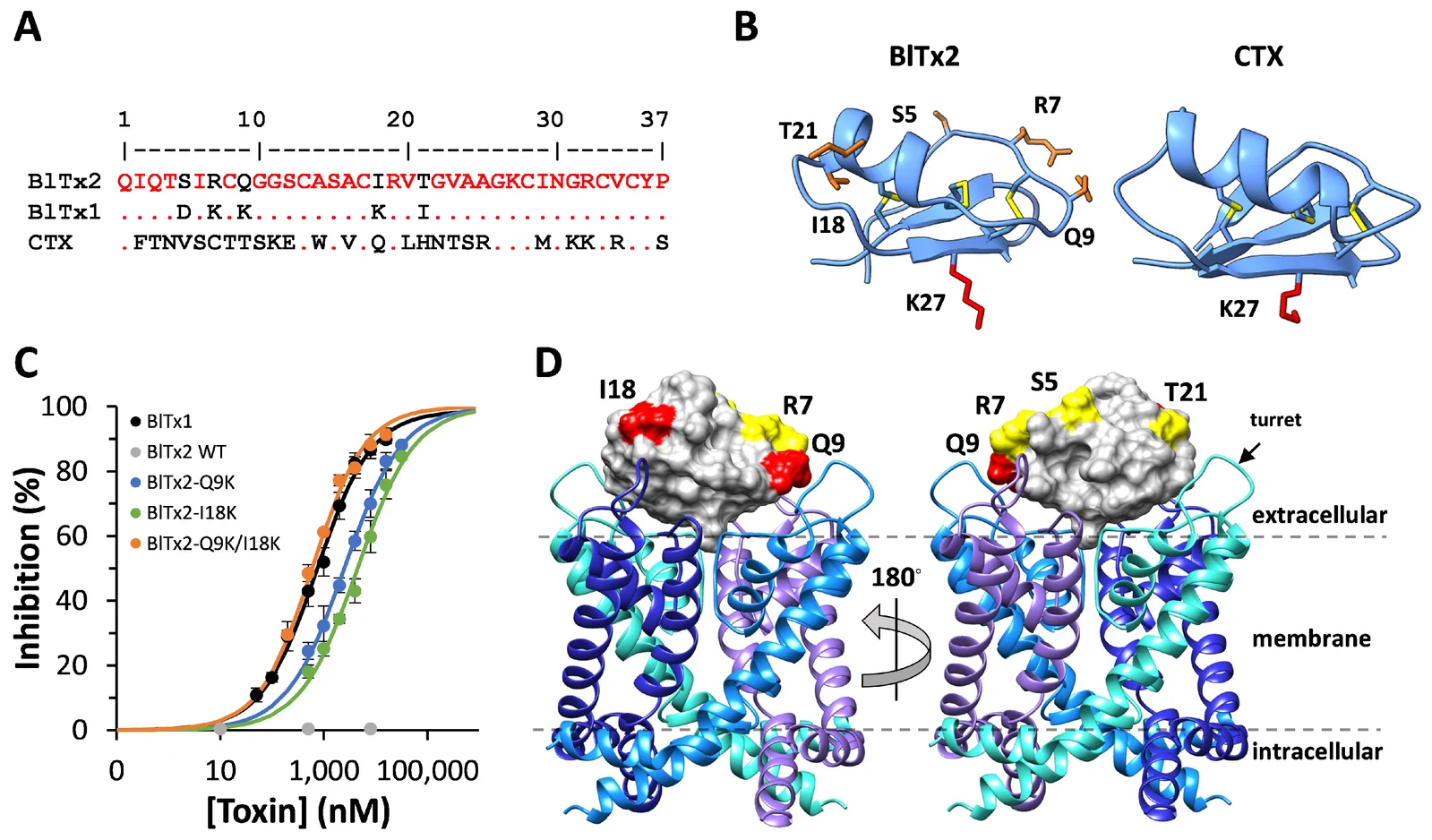
BlTx1-like substitutions that confer Kv1.2 inhibition on BlTx2. (**A**) Amino acid sequence alignment of BlTx2, BlTx1, and charybdotoxin (CTX). Dots indicate residues identical to BlTx2, whereas amino acid substitutions are shown explicitly. (**B**) Ribbon representations of the BlTx2 model (**left**), generated using PDB ID 6AY8 as a structural template, and CTX (**right**; PDB ID 2CRD, model 8). In BlTx2, residues that differ from the corresponding residues in BlTx1 are shown in orange. Disulfide bonds are shown in yellow, and the conserved pore-blocking Lys27 is shown in red in both toxins. (**C**) Concentration-response curves for BlTx1, BlTx2, and BlTx2-derived variants tested on Kv1.2. BlTx1 inhibited Kv1.2 with an IC_50_ of 727 ± 86 nM, whereas BlTx2 showed no detectable inhibition at concentrations up to 8000 nM. Among the BlTx2 variants, BlTx2-Q9K, BlTx2-I18K, and BlTx2-Q9K/I18K inhibited Kv1.2 with IC_50_ values of 2492 ± 103, 4488 ± 310, and 596 ± 45 nM, respectively. Data are shown as mean ± S.E.M. (*n* = 3–5), and solid lines represent fits to a modified Hill equation. (**D**) Structural model of BlTx2 bound to Kv1.2, shown in two side views rotated by 180°. Only the pore-forming TM5-TM6 region of each channel subunit is shown as ribbons, with each subunit colored differently. The channel structure was based on PDB ID 4JTA. BlTx2 is shown as a gray surface. Residues whose substitution with the corresponding BlTx1 residue conferred Kv1.2 inhibition, namely Gln9 and Ile18, are shown in red, whereas residues whose substitution did not confer Kv1.2 inhibition, namely Ser5, Arg7, and Thr21, are shown in yellow.

We previously reported the isolation of BlTx1, a paralog of BlTx2 that differs from it by only five residues [40] (Figure 3A). Despite substantial sequence differences from CTX, the modeled BlTx2 structure displays the characteristic α-KTx fold and a similarly positioned conserved Lys27 (Figure 3B). Although BlTx1 showed selectivity among Kv4 channel isoforms, it also inhibited Kv1.2, with an IC_50_ of 727 ± 86.2 nM (Figure 3C). We therefore used this difference between BlTx1 and BlTx2 to identify BlTx2 residues that contribute to its restricted activity profile among Kv channels. To this end, we generated five BlTx2 variants, each carrying one of the amino acid substitutions present in BlTx1, and tested their ability to inhibit Kv1.2 channels. Three variants, BlTx2-S5D, BlTx2-R7K, and BlTx2-T21I, showed no detectable inhibition of Kv1.2 at concentrations up to 8000 nM, similar to WT BlTx2. In contrast, BlTx2-Q9K and BlTx2-I18K produced detectable Kv1.2 inhibition, albeit with relatively low potency, with IC_50_ values of 2492 ± 103.1 nM and 4488 ± 310.3 nM, respectively. Both variants were significantly less potent than BlTx1 (*p* < 1 × 10^−3^; Figure 3C). The double variant BlTx2-Q9K/I18K inhibited Kv1.2 with an IC_50_ of 596 ± 44.9 nM, similar to that of BlTx1 (*p* = 0.42; Figure 3C). These findings indicate that lysine residues at positions 9 and 18 are major determinants of the ability of BlTx toxins to inhibit Kv1.2 channels, and that the absence of these lysine residues in BlTx2 contributes to its higher specificity for Kv4.1. Mapping the five BlTx1-like substitution sites onto the BlTx2-Kv1.2 model illustrates the locations of Gln9 and Ile18, whose substitutions conferred Kv1.2 activity, relative to the three positions that did not confer Kv1.2 activity (Figure 3D).

#### 2.2.2. A Kv1.2 Residue Facilitates Selective Interaction with BlTx Toxins

**Figure 4:**
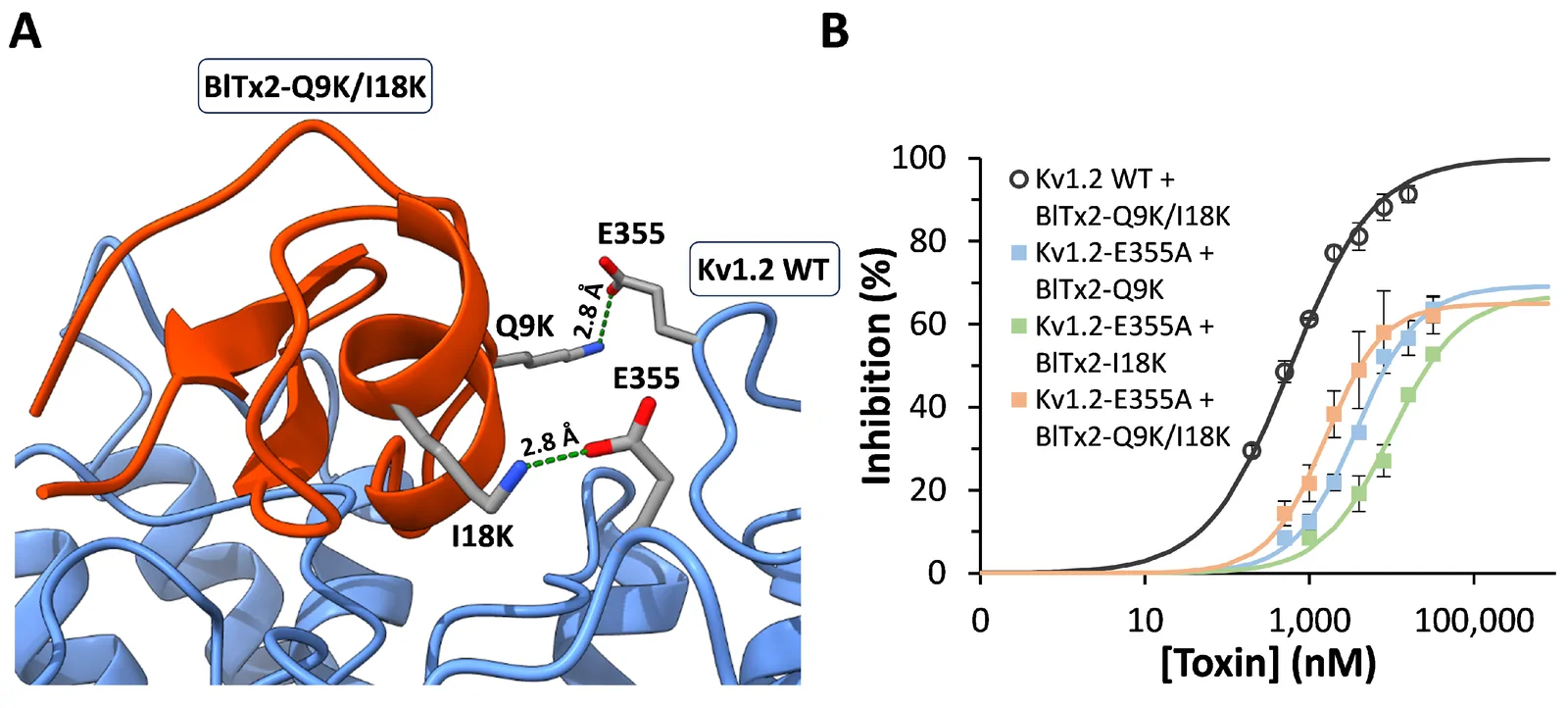
Predicted contribution of Kv1.2 Glu355 to the interaction with BlTx2 variants. (**A**) Representative ToxDock-refined structural model of BlTx2-Q9K/I18K positioned on the Kv1.2 outer vestibule, selected as described in Section 4.5. The toxin is shown in red and the channel in blue. In the model, Lys9 is positioned near Glu355 on one Kv1.2 subunit, whereas Lys18 is positioned near Glu355 on an adjacent subunit. Glu355 is equivalent to Asp351 in the Kv1.2-2.1 paddle chimera structure bound to charybdotoxin (PDB ID: 4JTA). This arrangement is consistent with two potential electrostatic contacts between the toxin and the channel. (**B**) Concentration-response curves showing inhibition of Kv1.2 WT and Kv1.2-E355A by BlTx2-Q9K/I18K, BlTx2-Q9K, and BlTx2-I18K. Data are shown as mean ± S.E.M. (*n* = 3–5), and solid lines represent fits to a modified Hill equation. IC_50_ values were 596 ± 45 nM for BlTx2-Q9K/I18K on Kv1.2 WT, and 1564 ± 100, 3748 ± 558, and 9794 ± 691 nM for BlTx2-Q9K/I18K, BlTx2-Q9K, and BlTx2-I18K on Kv1.2-E355A, respectively.

To examine how lysine residues at positions 9 and 18 may promote Kv1.2 inhibition, we inspected the structural model of Kv1.2 bound to BlTx2-Q9K/I18K, generated as described in Section 4.5. In this model, Lys9 and Lys18 are positioned near Glu355 residues contributed by two adjacent Kv1.2 subunits. This turret residue corresponds to Glu355 in the Kv1.2 sequence (NP_004965) and is equivalent to Asp351 in the charybdotoxin-bound Kv1.2-2.1 paddle chimera structure (PDB ID: 4JTA). This arrangement suggested that the binding of Kv1.2-active BlTx2 variants may be stabilized by electrostatic interactions, potentially including salt bridges, between the toxin’s Lys residues and the channel’s Glu355 residues at the outer vestibule (Figure 4A). To assess whether this arrangement was recurrent among plausible models, we measured the shortest distances between Lys9 or Lys18 NZ and either the Glu355 carboxylate oxygen or OE1 or OE2 in the 25 top-ranked ToxDock-refined complexes. Lys9 was positioned within 4.0 Å of a Glu355 carboxylate oxygen in 10 of 25 models, whereas Lys18 was positioned within 4.0 Å in 11 of 25 models. In 5 of 25 models, both Lys9-Glu355 and Lys18-Glu355 distances were between 2.8 and 3.0 Å. Thus, possible Lys-Glu electrostatic contacts were recurrent among the top-ranked models, supporting the plausibility of the proposed interaction. To test this prediction, Glu355 in Kv1.2 was substituted with alanine. The E355A mutant formed functional channels and did not differ significantly from WT Kv1.2 in either the voltage dependence of activation (V_1/2_ = −4.06 ± 1.61 and −5.22 ± 1.23 mV for WT and E355A, respectively) or the activation time constant (2.59 ± 0.30 and 2.32 ± 0.26 ms, respectively; Figure S2). The activity of the BlTx2 variants that inhibited Kv1.2 was reassessed on Kv1.2-E355A channels. The single variants BlTx2-Q9K and BlTx2-I18K showed reduced potency on Kv1.2-E355A, with apparent IC_50_ values of 3748 ± 557 nM and 9794 ± 691 nM, respectively. These values were significantly higher than those measured for the same variants on WT Kv1.2 (*p* < 1 × 10^−3^ for each comparison; Figure 4B). Similarly, the apparent potency of the double variant BlTx2-Q9K/I18K for Kv1.2-E355A was reduced by approximately threefold compared with WT Kv1.2 (IC_50_ = 1564 ± 100 nM, *p* < 1 × 10^−3^; Table 1). On both WT Kv1.2 and Kv1.2-E355A, BlTx2-Q9K/I18K was more potent than either single variant, and BlTx2-Q9K was more potent than BlTx2-I18K (*p* < 1 × 10^−3^ for each comparison; Table 1).

Together, these results support a functional contribution of both toxin lysine residues and Kv1.2 Glu355 to toxin inhibition, consistent with the proximity predicted by the structural model. The stronger effect observed for BlTx2-I18K suggests that Lys18 may depend more strongly on the local environment involving Glu355, whereas Lys9 may also be influenced by other residues in the Kv1.2 outer vestibule.

In addition to the reduction in apparent potency, all three variants showed a marked decrease in maximal blockade of Kv1.2-E355A. Whereas these toxins produced nearly complete inhibition of WT Kv1.2, maximal inhibition of Kv1.2-E355A was limited to approximately 65–70% for all variants tested (*p* < 1 × 10^−3^ for each comparison; Figure 4B).

### 2.3. Molecular Determinants of Kv4.1 That Allow Selective Interaction with BlTx2

**Figure 5:**
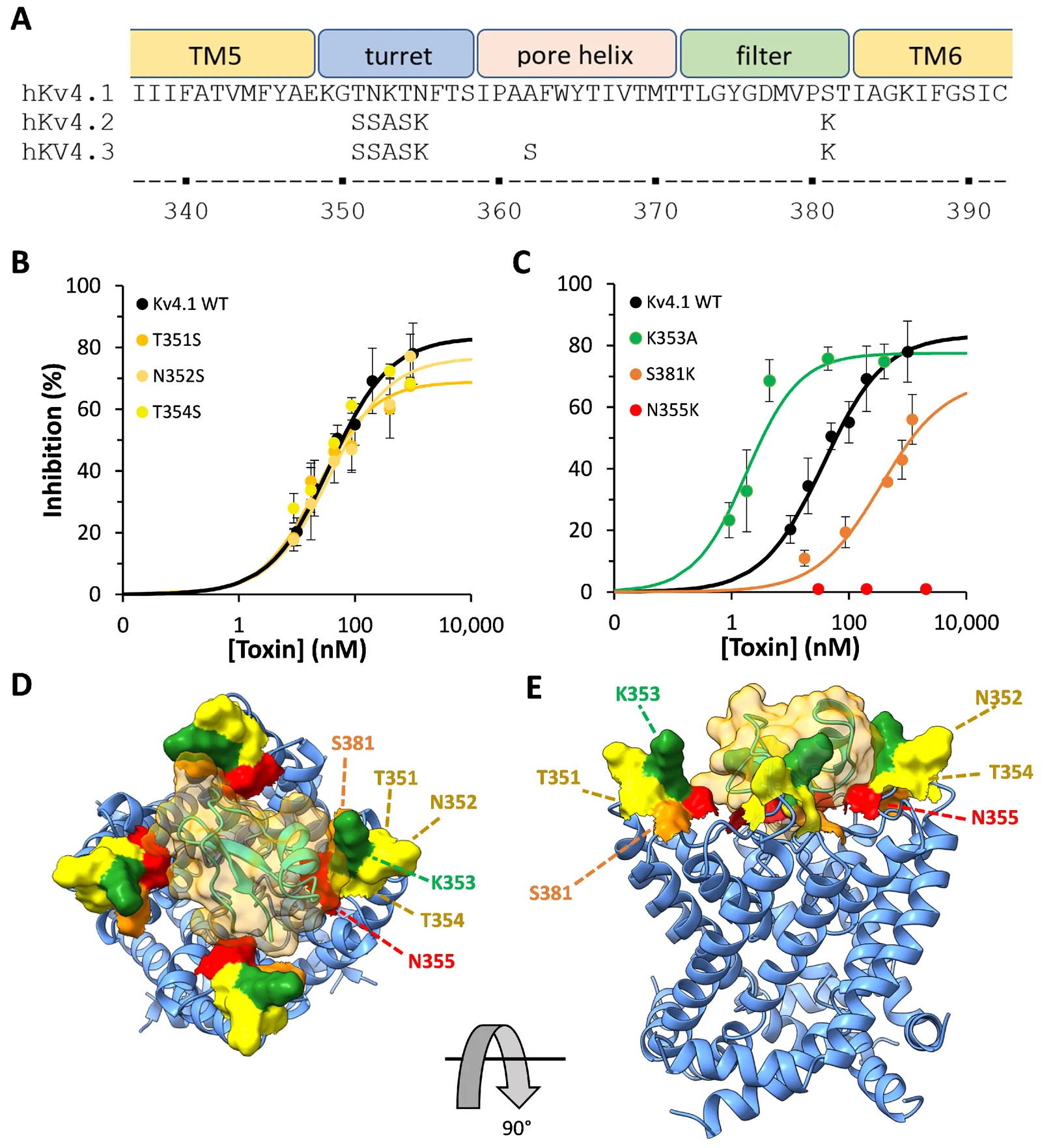
Locating the BlTx2 binding site on Kv4.1. (**A**) Comparison of the amino acid sequences of Kv4.1, Kv4.2, and Kv4.3 in the pore region, including TM5, the turret, the pore helix, the selectivity filter, and TM6, as indicated. (**B**) Concentration-response curves for Kv4.1 mutants that did not significantly alter BlTx2 sensitivity. Data are shown as mean ± S.E.M. (*n* = 3–5), and solid lines represent fits to a modified Hill equation. The IC_50_ values for T351S, N352S, and T354S were 22.2 ± 4.5, 36.9 ± 13.2, and 18.8 ± 4.4 nM, respectively. (**C**) Concentration-response curves for K353A, S381K, and N355K, measured as in (**B**). K353A increased BlTx2 potency (IC_50_ = 1.8 ± 0.3 nM, green), whereas S381K reduced BlTx2 potency (IC_50_ = 350 ± 56 nM, orange). N355K produced no detectable inhibition at concentrations up to 2000 nM (red). (**D**,**E**) Structural model of BlTx2 bound to Kv4.1, generated as described in Section 4.5. The Kv4.1 channel is shown as blue ribbons, and BlTx2 is shown as an orange surface. The model is displayed from top (**D**) and side (**E**) views. The surfaces of channel residues that differ between Kv4.1 and the corresponding positions in Kv4.2 and Kv4.3 are shown and colored according to the effect of their substitution on BlTx2 inhibition: yellow, no significant effect; green, increased potency; orange, reduced potency; red, no detectable inhibition at the tested toxin concentrations.

To identify the channel determinants underlying the isoform selectivity of BlTx2 for Kv4.1, we aligned the sequences of Kv4.1, Kv4.2, and Kv4.3 within the putative toxin-binding region. This region includes the extracellular ends of TM5 and TM6, the turret, and the selectivity filter. The alignment identified six residues that differ between Kv4.1 and the corresponding positions in Kv4.2 and Kv4.3, which are identical to each other at these sites (Figure 5A). Each of these six Kv4.1 residues was individually substituted with the residue present at the corresponding position in Kv4.2 and Kv4.3. All six mutants formed functional channels and did not differ significantly from WT Kv4.1 in their activation V_1/2_ values (Figure S3). Mutations T351S, N352S, N355K, and S381K also had no significant effect on the Kv4.1 inactivation time constant. Mutations K353A and T354S produced modest but statistically significant increases in the inactivation time constant (49.4 ± 2.8, 66.8 ± 3.6, and 65.8 ± 3.8 ms for WT, K353A, and T354S, respectively; Figure S3).

The effect of these six mutations on BlTx2 inhibition was then examined. Three substitutions, T351S, N352S, and T354S, had little or no effect on BlTx2 sensitivity. Their IC_50_ values were 22.2 ± 4.5, 36.9 ± 13.2, and 18.8 ± 4.4 nM, respectively, and did not differ significantly from WT Kv4.1 (*p* = 0.35, 0.90, and 0.09, respectively; Figure 5B). In contrast, the remaining three Kv4.1-to-Kv4.2/Kv4.3 substitutions produced marked effects. Replacement of Lys353 with alanine substantially increased BlTx2 potency, shifting the IC_50_ to 1.8 ± 0.3 nM, indicating that the native lysine at this position limits BlTx2 inhibition. Conversely, introduction of lysine at position 381 reduced potency, with an IC_50_ of 350 ± 56 nM, and introduction of lysine at position 355 abolished detectable inhibition at concentrations up to 2000 nM (*p* < 1 × 10^−3^ for each comparison with WT Kv4.1; Figure 5C). These results indicate that introducing lysine at positions 355 and 381 impairs BlTx2 inhibition, with the effect being most pronounced at position 355.

Mapping these residues onto the structural model showed that the three substitutions with little or no functional effect, T351S, N352S, and T354S, are located at positions oriented away from the predicted toxin-binding interface, whereas the positions at which substitution markedly altered BlTx2 inhibition, Lys353, Asn355, and Ser381, cluster in the extracellular pore region facing the bound toxin (Figure 5D,E). Together, these results indicate that the identity of residues at Kv4.1 positions 353, 355, and 381 strongly influences BlTx2 sensitivity. Specifically, replacement of Lys353 with alanine increased BlTx2 potency, whereas introduction of lysine at positions 381 or 355, corresponding to the residues present at these positions in Kv4.2 and Kv4.3, reduced or abolished detectable inhibition. Thus, Kv4.1 selectivity appears to reflect the absence of Kv4.2/Kv4.3-like lysine residues that interfere with BlTx2 inhibition, together with other features of the Kv4.1 outer vestibule that permit productive toxin binding.

### 2.4. Structure-Guided Mutational Mapping of the BlTx2-Kv4.1 Interaction Interface

**Figure 6:**
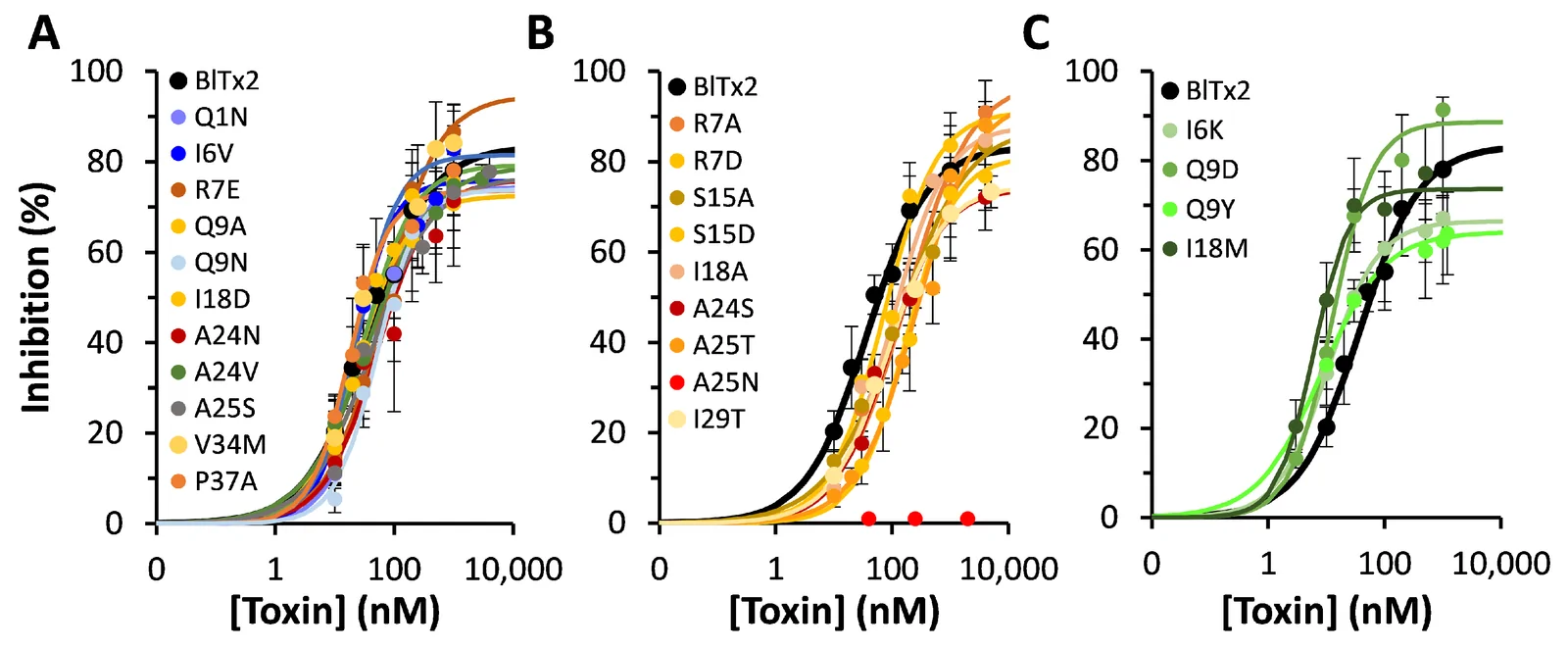
Inhibitory potency of BlTx2 variants on Kv4.1 channels. BlTx2 variants were tested as described for Figure 1 (mean ± S.E.M., *n* = 3–5). Solid lines represent fits of the data to a modified Hill equation. IC_50_ values for all variants are listed in Table 2. Statistical significance was determined relative to WT BlTx2 as described in Section 4.7. (**A**) Concentration-response curves for BlTx2 variants that exhibited no significant effect on Kv4.1 inhibition. (**B**) Concentration-response curves for BlTx2 variants with significantly reduced inhibitory potency. The A25N mutant displayed no detectable inhibition at concentrations up to 2 μM. (**C**) Concentration-response curves for BlTx2 variants with significantly enhanced inhibitory potency.

To further define the Kv4.1-interacting surface of BlTx2, we used the structural model described in Section 4.5 to guide a broader mutational analysis of the toxin. Candidate substitutions were selected based on the predicted position of toxin residues relative to the Kv4.1 outer vestibule, including their potential contributions to steric interactions, hydrophobic packing, and electrostatic complementarity. Each variant was tested for concentration-dependent inhibition of Kv4.1, and the resulting IC_50_ values were compared with those of WT BlTx2 (Figure 6, Table 2).

Eleven substitutions had little or no effect on the apparent potency of BlTx2 toward Kv4.1 (Figure 6A, Table 2). In contrast, nine substitutions, including N30A, substantially reduced toxin potency. A25N abolished detectable inhibition within the tested concentration range (Figure 6B, Table 2), as did K27A and Y36A in the initial analysis of the conserved α-KTx functional residues (Figure 1, Table 2).

**Figure 7:**
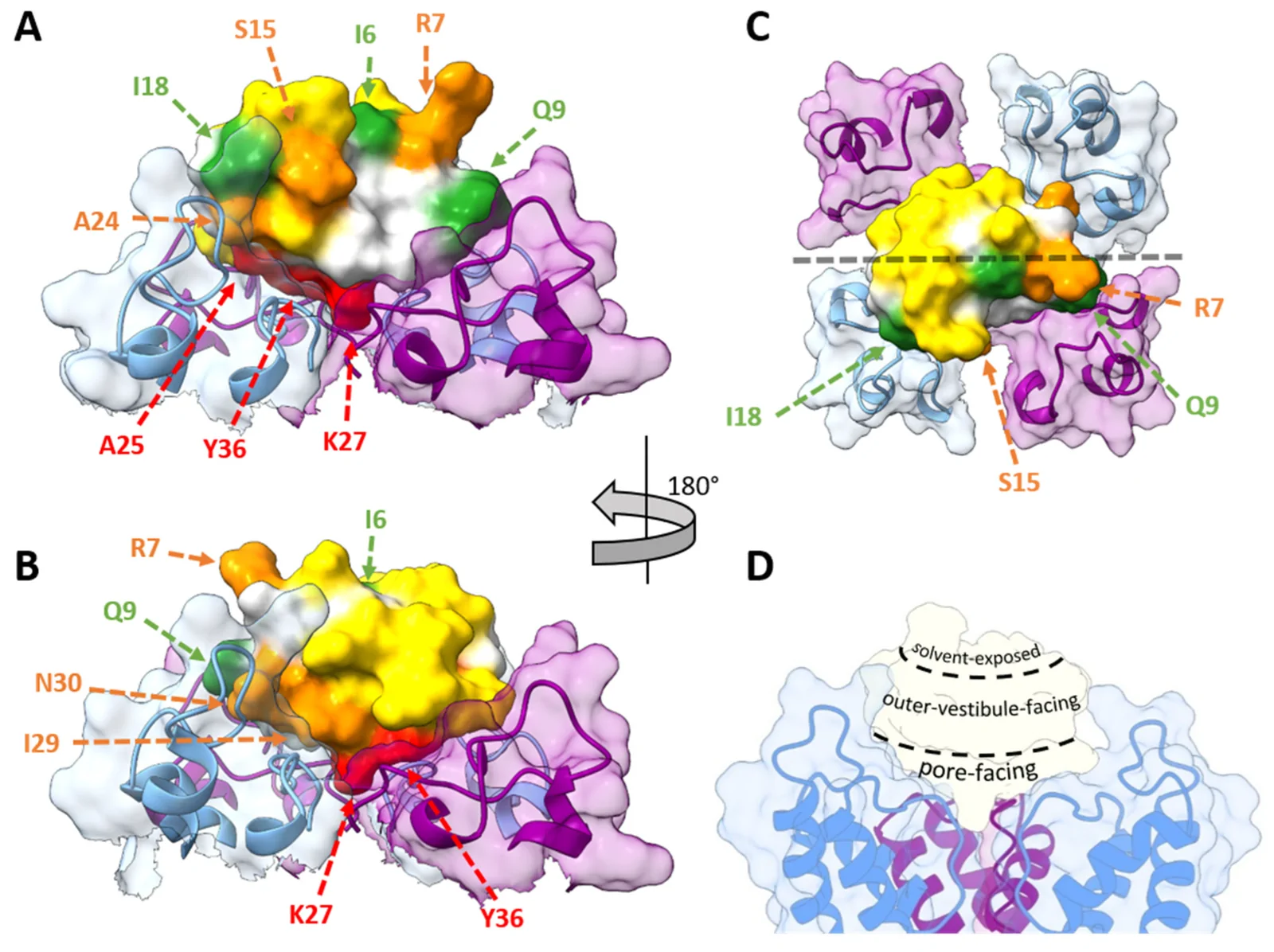
Structural mapping of functionally important BlTx2 residues on the predicted BlTx2-Kv4.1 complex. Residues subjected to mutational analysis are mapped onto the structural model of BlTx2 bound to Kv4.1. BlTx2 is shown as a surface and colored according to the strongest functional effect observed among the substitutions tested at each position: red, substitutions that abolished or severely impaired Kv4.1 inhibition; orange, substitutions that significantly reduced inhibitory potency; green, substitutions that significantly enhanced inhibitory potency; yellow, substitutions with little or no effect on inhibitory potency; gray, residues that were not mutated. Only the toxin-binding region of the channel is shown. Kv4.1 is shown as a ribbon with a transparent surface. (**A**,**B**) Opposite side views of the complex, viewed approximately parallel to the membrane plane. Residues whose substitution reduced or abolished inhibition cluster predominantly on the predicted pore-facing surface of the toxin, whereas residues whose substitution enhanced inhibition, or caused only a moderate reduction in potency, are located more peripherally, toward the outer region of the predicted binding interface and the upper pore vestibule. (**C**) Top view of the complex from the extracellular side. The dashed line is used as a visual reference to illustrate the asymmetric position of BlTx2 relative to the channel pore. (**D**) The toxin surface was divided into three theoretical functional regions according to their orientation relative to the channel: pore-facing, outer-vestibule-facing, and solvent-exposed regions. The toxin surface is shown in yellow, and the channel surface is shown in transparent blue.

Conversely, four substitutions enhanced toxin potency. The mutants I6K, Q9D, Q9Y, and I18M exhibited IC_50_ values of 10.7 ± 2.1, 12.6 ± 1.3, 8.01 ± 1.78, and 5.98 ± 0.89 nM, respectively (Figure 6C, Table 2). Mapping these effects onto the structural model revealed a clear spatial segregation of functionally important residues (Figure 7). Residues whose substitution reduced or abolished Kv4.1 inhibition clustered predominantly on the predicted pore-facing surface of the toxin. In contrast, residues whose substitution enhanced toxin potency were located more peripherally, toward the outer region of the predicted binding interface and the upper portion of the pore vestibule. These observations provide additional support for the proposed BlTx2-Kv4.1 interaction model.

For several BlTx2 mutants, namely I2E, Q3E, S5D, A14E, R19Q, V20E, and T21I, activity was assessed by comparing inhibition at 50 nM with that produced by WT BlTx2. All of these mutants retained WT-like inhibitory activity, with no significant difference from WT BlTx2, as determined by one-way ANOVA followed by Dunnett’s multiple-comparison test. The structural model predicts an asymmetric interaction between BlTx2 and the channel, with one side of the toxin, including residues such as Gln9, Ser15, and Ile18, positioned near two adjacent Kv4.1 subunits. In contrast, the opposite side of the toxin is distant from the channel turret and is not predicted to contribute substantially to the interaction. Consistent with this model, substitutions on this distal side of the toxin did not measurably affect Kv4.1 inhibition (Figure 7C).

## 3. Discussion

The present study identifies the molecular determinants that allow the scorpion α-KTx15 peptide BlTx2 to act as a selective inhibitor of Kv4.1 among the Kv channels tested. The specific contribution of Kv4.1 to fast transient A-type potassium currents has been difficult to resolve because most available pharmacological tools either block several Kv4 isoforms or affect additional potassium channel families. BlTx2, therefore, provides a rare opportunity to examine Kv4.1 function with acute pharmacological selectivity. Here, by combining toxin mutagenesis, channel mutagenesis, tandem-channel analysis, functional electrophysiology, and structural modeling, we show that BlTx2 employs the conserved α-KTx pore-blocking strategy. Still, its selectivity is determined by a more restricted set of toxin and channel residues in the channel’s outer vestibule.

### 3.1. BlTx2 Binds to Kv4.1 Using the Known Mechanism of α-KTx Toxins

A central conclusion from this work is that BlTx2 blocks Kv4.1 through a mechanism related to the canonical α-KTx pore-blocking mode. The alanine substitutions K27A and Y36A abolished detectable inhibition, whereas N30A markedly reduced potency (Figure 1). These residues correspond to the classical functional surface of many α-KTx toxins, in which a conserved lysine protrudes into the external mouth of the pore and neighboring residues stabilize the toxin-channel complex [18,19,20,25,41,42]. Thus, although BlTx2 is unusually selective among Kv4 channels, its basic blocking mechanism does not appear to be unique. Rather, it represents a Kv4.1-selective implementation of the general α-KTx pore-blocking architecture. This distinction is important because it separates two mechanistic layers: a conserved pore-blocking core shared with other α-KTx toxins and peripheral or channel-specific determinants that tune selectivity.

The tandem-dimer experiment (Figure 2) is consistent with a contribution of multiple channel subunits to high-affinity BlTx2 inhibition. In the alternating Kv4.1/Kv4.3 tandem chimera, two Kv4.1 subunits are present, but they are expected to be positioned diagonally rather than adjacent to one another. The marked reduction in BlTx2 potency on this channel supports the idea that a single Kv4.1 subunit, or two non-adjacent Kv4.1 subunits, is insufficient for high-affinity inhibition. Instead, the data support a model in which adjacent Kv4.1 subunits together form the appropriate extracellular vestibule recognized by BlTx2. This conclusion agrees with the established view that α-KTx toxins bind to a receptor surface assembled from several subunits of the tetrameric channel [15,19,25,28,29,43]. Consistent with that notion, we found that mutations that affect toxin activity are not evenly distributed across the toxin surface but are clustered asymmetrically on one side (Figure 7C). Nevertheless, because tandem constructs can alter local channel geometry, assembly, or toxin accessibility, this result should be regarded as supportive rather than definitive evidence of a multi-subunit binding surface, and it does not establish the exact subunit contacts.

### 3.2. Kv4.1 Mutagenesis Identifies Channel Positions That Shape BlTx2 Selectivity Within the Kv4 Subfamily

The channel-mutagenesis experiments identify three Kv4.1 pore-region positions, 353, 355, and 381, at which Kv4.1-to-Kv4.2/Kv4.3 substitutions strongly alter BlTx2 sensitivity (Figure 5). Among the six positions that differ between Kv4.1 and the corresponding positions in Kv4.2 and Kv4.3, the substitutions T351S, N352S, and T354S, which are predicted to face away from the toxin, had little or no effect on BlTx2 inhibition. In contrast, substitutions at three positions predicted to face the toxin had a marked effect on functional inhibition. K353A increased toxin potency, whereas S381K strongly reduced potency. The effect of K353A is informative because it suggests that Lys353 may impose an unfavorable electrostatic or steric constraint in the native channel, and that BlTx2 sensitivity is determined by a balance of favorable and unfavorable local interactions rather than by a single dominant contact. The most informative substitution was N355K, which eliminated detectable inhibition across the tested concentration range. These results suggest that the lysine residue present at the corresponding position in Kv4.2 and Kv4.3 is incompatible with BlTx2 inhibition and may be a major contributor to their resistance to BlTx2.

### 3.3. Defining the Active Surface of BlTx2

Inspection of our BlTx2-Kv4.1 model, generated using the Kv1.2-2.1 paddle chimera channel-charybdotoxin structure as a template (PDB ID: 4JTA), provides a useful framework for interpreting the BlTx2 mutational data. In this model, the BlTx2 surface can be divided into three regions: a pore-facing region, containing residues oriented toward the central pore; an outer-vestibule-facing region, containing residues positioned near the upper outer vestibule and turret; and a solvent-exposed region, containing residues oriented away from the channel and exposed primarily to the extracellular solution (Figure 7D). When viewed from the extracellular side, the projected BlTx2 footprint can also be divided into two lateral sectors relative to a pore-centered reference axis: an outer-vestibule-proximal sector, positioned closer to the Kv4.1 outer pore vestibule and turret region, and an outer-vestibule-distal sector, positioned farther from this region and more exposed to the extracellular solution (Figure 7C). We use this structural categorization below to discuss how different groups of BlTx2 residues contribute to Kv4.1 inhibition.

The broader toxin-mutagenesis screen extends this picture from the channel side to the toxin surface. Substitutions that impaired or abolished Kv4.1 inhibition clustered primarily on the predicted pore-facing surface of BlTx2 (Figure 7A,B). This group includes the conserved, functionally important residues Lys27, Asn30, and Tyr36, as well as Ala25, Ala24, and Ile29, a distribution consistent with the proposed orientation of the toxin over the channel pore entry. In the outer-vestibule-facing region, substitutions of two residues, Arg7 and Ser15, reduced activity, whereas substitutions of Gln9 and Ile18 increased toxin potency and are discussed below. Furthermore, when viewed from the extracellular side, substitutions that measurably affected Kv4.1 inhibition were concentrated in the outer-vestibule-proximal sector, whereas substitutions in the outer-vestibule-distal sector had no significant effect on Kv4.1 inhibition (Figure 7C).

**Figure 8:**
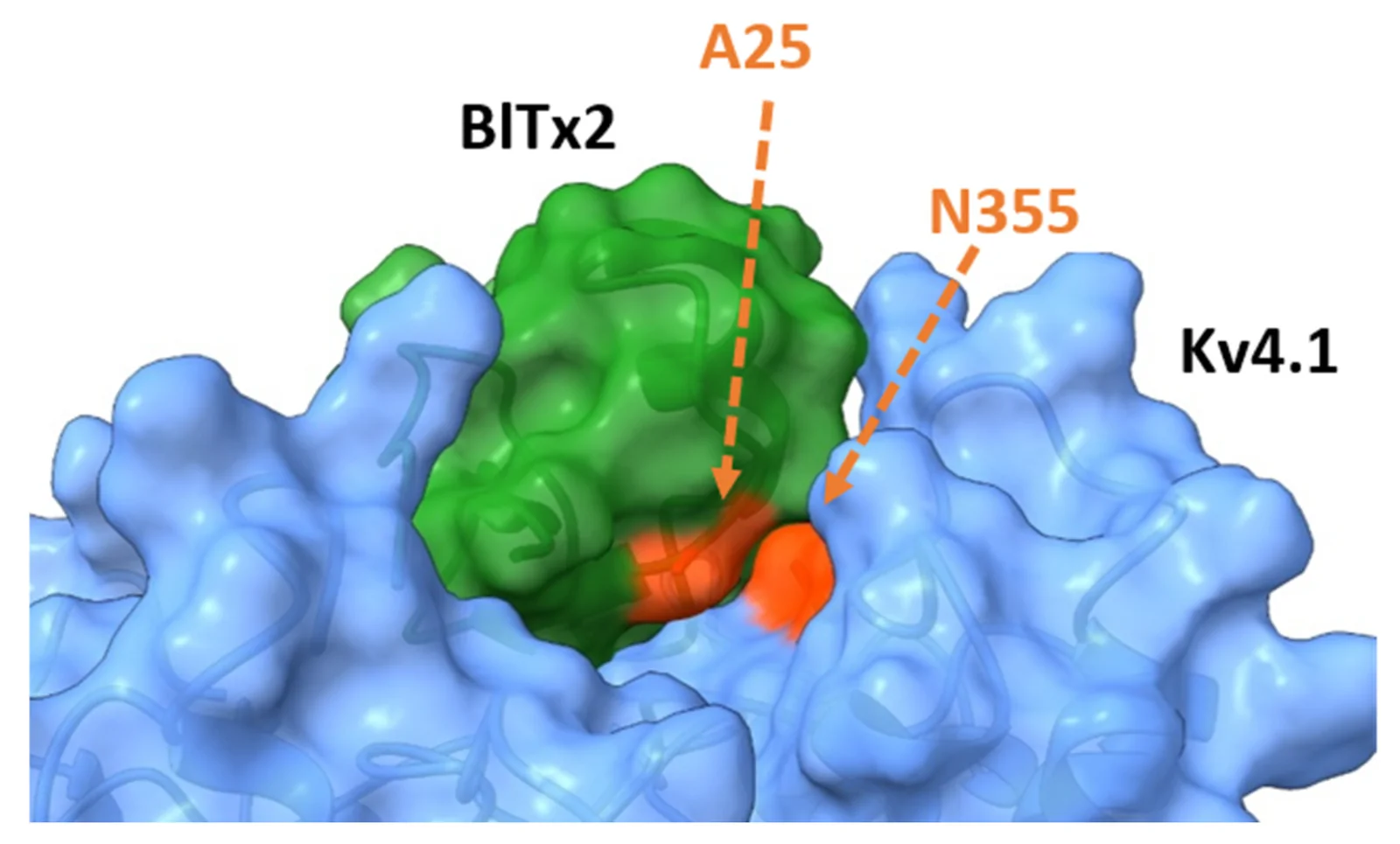
Predicted proximity between BlTx2 Ala25 and Kv4.1 Asn355. Structural model of BlTx2 bound to the Kv4.1 outer vestibule. BlTx2 is shown as a green surface, and Kv4.1 is shown as a blue surface with the underlying ribbon. BlTx2 Ala25 and Kv4.1 Asn355 are highlighted in orange. In this model, Ala25 is positioned near Asn355.

The complete loss of activity caused by A25N is especially consistent with a tightly constrained local interface, possibly involving steric interactions between the toxin at position 25 and the channel (Figure 8). To support this hypothesis, the graded functional effects of substitutions at Ala25 correlated with the expected increase in side-chain volume from Ala (67 Å^3^; IC_50_ = 35.6 ± 7.5 nM) to Ser (73 Å^3^; IC_50_ = 41.5 ± 11.7 nM), Thr (93 Å^3^; IC_50_ = 260 ± 28 nM), and Asn (96 Å^3^; no detectable inhibition) (Table 2) [44]. In the WT BlTx2-Kv4.1 model, the Cβ atom of BlTx2 Ala25 is positioned 4.4 Å from the nearest side-chain oxygen of Kv4.1 Asn355, placing the two residues in proximity within the modeled interface. Modeling the A25N substitution showed substantial dependence on side-chain rotamer: some conformations produced sterically incompatible short contacts with Asn355, whereas others could be accommodated without an obvious severe clash. Thus, the model does not demonstrate an obligatory steric collision, but it indicates that enlargement of the side chain at toxin position 25 could restrict the available conformational space or require local rearrangement of the toxin-channel interface. Consistent with this interpretation, substitution of Kv4.1 Asn355 with lysine, which increases side-chain volume from 96 Å^3^ to 135 Å^3^ [44] and also alters charge and side-chain geometry, abolished detectable BlTx2 inhibition (Figure 5). Taken together, the reciprocal functional effects of increasing side-chain bulk at toxin position 25 and channel position 355, together with their close spatial proximity in the model, support the interpretation that this region of the interface is sterically constrained. However, the observed effects cannot be attributed uniquely to steric interference, as changes in polarity, hydrogen-bonding capacity, electrostatics, hydration, side-chain geometry, or local conformational rearrangement may also contribute.

Other physicochemical factors, including changes in hydration at the toxin-channel interface, may also contribute to the effects of these substitutions. Such effects could involve rearrangement or displacement of interfacial water molecules, but they cannot be evaluated quantitatively from the present structural models and therefore remain speculative.

**Figure 9:**
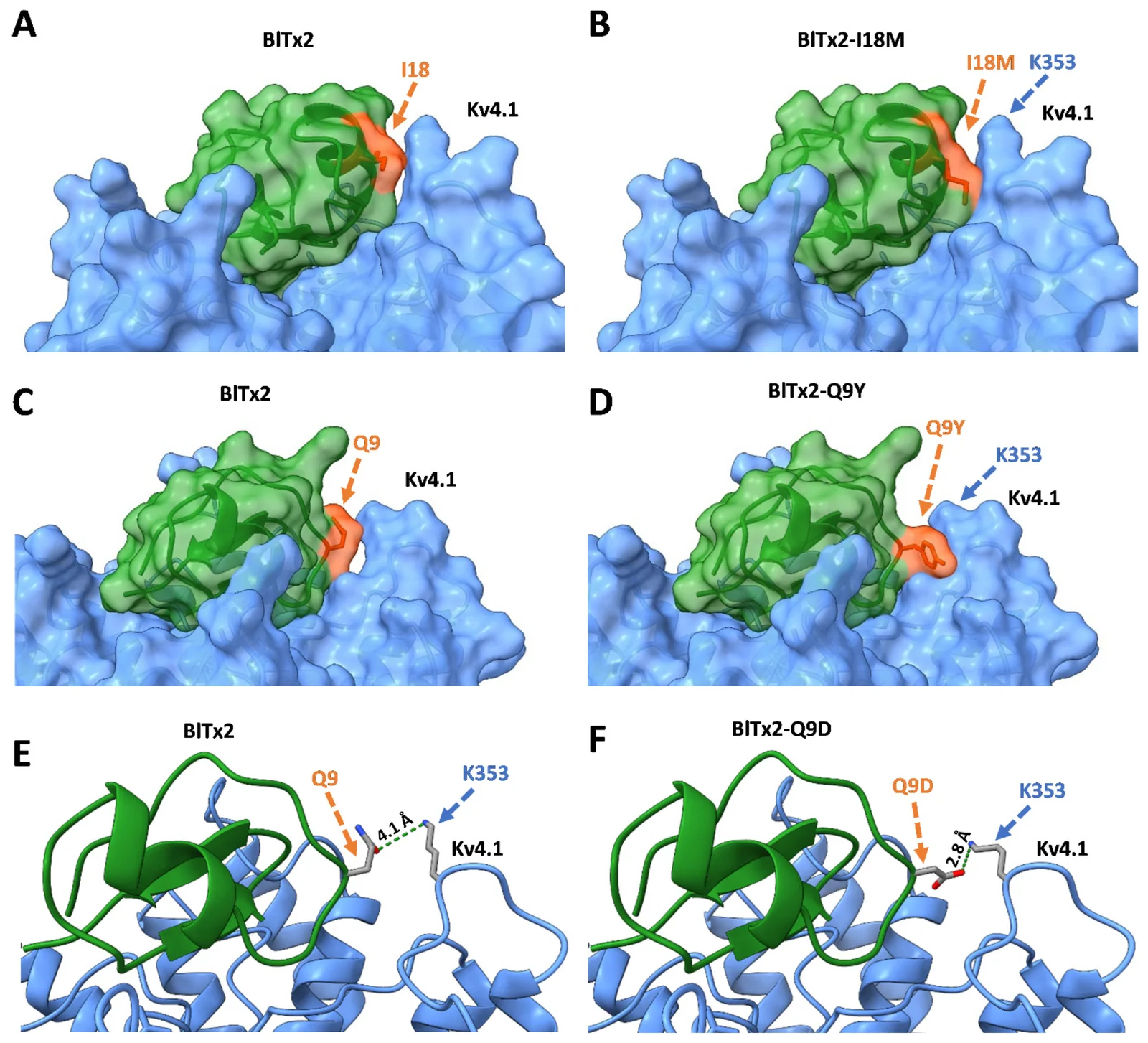
Models illustrating the proposed structural basis for the enhanced potency of BlTx2-I18M, BlTx2-Q9Y, and BlTx2-Q9D. (**A**–**D**) Surface representations of BlTx2 bound to Kv4.1. The toxin is shown in green and the channel in blue. Residues at toxin positions 18 or 9 are shown in orange. WT BlTx2 is shown for comparison in (**A**,**C**), whereas BlTx2-I18M and BlTx2-Q9Y are shown in (**B**,**D**), respectively. In the models, Met18 in BlTx2-I18M and Tyr9 in BlTx2-Q9Y are positioned near a local pocket adjacent to Kv4.1 Lys353 residues from neighboring subunits. These substitutions may permit additional hydrophobic or aromatic interactions that contribute to the increased potency of the mutant toxins. (**E**) Ribbon representation of the WT BlTx2-Kv4.1 complex. Panel (**E**) shows the same ToxDock-derived WT complex as panel (**C**), displayed in a different representation to highlight the local environment of Gln9 and Lys353. (**F**) Ribbon representation of BlTx2-Q9D bound to Kv4.1. Toxin residue 9 and Kv4.1 Lys353 are shown as sticks and colored by element. In the WT model, the Gln9 OE1 atom is positioned near the NZ atom of Kv4.1 Lys353 (4.1 Å). In the BlTx2-Q9D model, the Asp9 carboxylate is positioned closer to Lys353, with the shortest carboxylate oxygen-NZ distance compatible with a possible salt bridge.

In contrast, substitutions that enhanced potency were located in the outer-vestibule-facing region (Q9D, Q9Y, and I18M) and at a solvent-exposed position (I6K; Figure 7). These results suggest that BlTx2 is not maximally optimized for Kv4.1 inhibition at all positions. For Q9Y and I18M, we hypothesize that the increased potency results from the introduction or strengthening of hydrophobic interactions between the substituted toxin residues and a local pocket near Kv4.1 Lys353, formed by the surrounding backbone environment and the aliphatic portion of the Lys353 side chain in adjacent channel subunits (Figure 9A–D). For BlTx2-Q9D, the model suggests that substitution of Gln9 by Asp may position the negatively charged Asp9 carboxylate closer to Lys353, with the shortest carboxylate oxygen-NZ distance compatible with a possible salt bridge (Figure 9E,F). Ile6, in contrast, is predicted to face away from the channel, with no obvious proximity to channel residues. The enhancing effect of I6K is consistent with previous observations for the toxin Discrepin (α-KTx15.6), in which introduction of a Lys at position 6 increased the block of A-type/Kv4 currents [38,39]. Because Ile6 is not predicted to contact the channel directly, its effect is unlikely to arise from a simple local toxin-channel contact. Structural models generated for the toxin variants that affected activity suggest that I6K may produce a somewhat larger deviation in the predicted toxin backbone conformation, whereas the other substitutions largely preserve the WT-like fold (Figure S4). Thus, we speculate that the effect of I6K on toxin-channel binding may reflect an indirect contribution through altered toxin conformation, local dynamics, electrostatic surface potential, hydration, or positioning of residues that directly contact the channel.

### 3.4. Structural Basis for BlTx Selectivity Among Kv Channels

A comparison between BlTx1 and BlTx2 provides a particularly useful example of how small differences in toxin sequence can alter channel selectivity. BlTx1 differs from BlTx2 by only five residues, yet it inhibits both Kv4.1 and Kv1.2, whereas BlTx2 is restricted to Kv4.1 among the tested channels. Introducing BlTx1-like lysines at positions 9 and 18 into BlTx2 conferred Kv1.2 activity, and the double variant Q9K/I18K inhibited Kv1.2 with potency similar to BlTx1 (Figure 3). Thus, Kv1.2 cross-reactivity can be generated by a small number of peripheral substitutions without changing the canonical pore-blocking core. The ability of each of the two single lysine substitutions to produce weaker Kv1.2 inhibition, and of the double variant to recapitulate BlTx1-like potency, indicates that positions 9 and 18 both contribute to Kv1.2 inhibition.

The Kv1.2-E355A results support a functional role for Glu355 in Kv1.2 inhibition by the Kv1.2-active BlTx2 variants (Figure 4). In the structural model, Lys9 and Lys18 are positioned near Glu355 residues contributed by adjacent Kv1.2 subunits. Consistent with this model-derived prediction, substitution of Glu355 with alanine reduced the apparent potency of Q9K, I18K, and Q9K/I18K. E355A did not significantly alter the voltage dependence of activation or activation kinetics, arguing against a major disruption of channel function. However, the mutation also reduced the maximum level of toxin inhibition, suggesting it may affect the local toxin-binding environment or, more broadly, the efficacy of toxin-mediated pore blocking. Thus, although the functional data are consistent with the proximity predicted by the structural model, they do not establish a direct Lys-Glu interaction.

The predicted involvement of toxin residues 9 and 18 in the BlTx2-Kv4.1 and BlTx2-Kv1.2 models is consistent with the functional effects of substitutions at these positions. This agreement supports using the structural models as frameworks for interpreting the electrophysiological data, while recognizing that the present experiments do not directly establish the modeled residue-residue contacts and binding orientations.

### 3.5. Evolutionary Considerations

**Figure 10:**
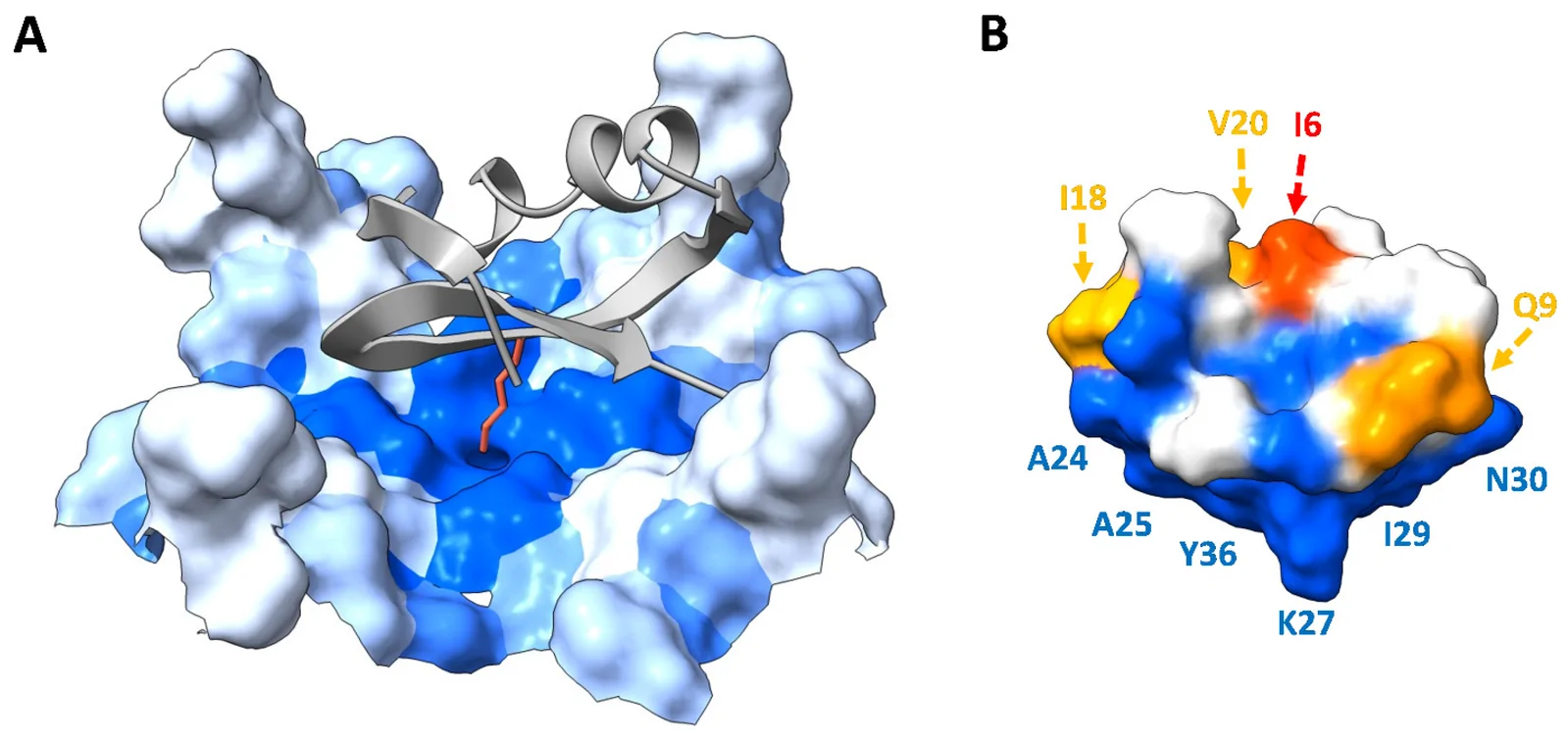
Conservation of Kv channel and α-KTx15 toxin residues. (**A**) Surface representation of the outer vestibule of Kv1.2, based on the Kv1.2-charybdotoxin structure, PDB 4JTA. Charybdotoxin is shown as a ribbon, with Lys27 shown in red. Only Kv1.2 residues facing the toxin are shown. Human Kv channels from subfamilies Kv1-Kv9 were aligned, and the degree of residue identity at each position was calculated. Kv1.2 residues are color-coded according to their conservation level, using a gradient from dark blue, indicating highly conserved residues, to very light blue, indicating weakly conserved residues. (**B**) Surface representation of the BlTx2 model, as described in Figure 3. Residues are colored according to the predicted selection pressures acting on the α-KTx15 toxin family, as determined by dN/dS analysis (Section 4.6). Residues under strong purifying selection are colored blue, residues predicted to be under strong diversifying selection are colored red, residues considered to show moderate evidence for diversifying selection are colored orange, and residues consistent with neutral evolution are colored white.

Diversifying selection has been proposed to contribute to the evolution of toxins in several venomous animal groups [45,46,47,48,49,50,51,52,53,54], whereas purifying selection can preserve residues important for toxin structure or function [55,56]. These opposing selective pressures may therefore help maintain conserved structural and functional determinants while allowing diversification of other toxin surfaces. Inspection of sequence conservation in Kv channels at the predicted toxin-binding region revealed a gradient from strong conservation near the extracellular pore and selectivity filter to lower conservation toward the outer vestibule and turret (Figure 10A). A related pattern was observed in α-KTx15 toxins (Figure 10B). Several residues under purifying selection, including Ala25, Lys27, Asn30, and Tyr36, are located on the pore-facing toxin surface, whereas more variable residues, including Gln9 and Ile18, are positioned toward the outer-vestibule-facing region. Ile6 and Val20 are predicted to face away from the channel. These patterns are broadly consistent with our functional results. Substitutions at several conserved pore-facing positions either abolished detectable activity or reduced potency, whereas substitutions at more peripheral positions produced a wider range of effects, including increased potency and altered channel selectivity. For example, substitutions at Gln9 and Ile18 altered BlTx2 activity toward both Kv4.1 and Kv1.2, suggesting that these positions may contribute to target-specific interactions. Similarly, Arg18 of BmTx3 has been implicated in hERG inhibition through a functional surface distinct from the classical Lys27/Tyr36-containing pore-blocking surface [37,57]. Taken together, our results suggest that the conserved pore-facing surface of α-KTx15 toxins may primarily support interaction with the conserved outer pore of Kv channels, whereas more variable peripheral residues may contribute to differences in potency and selectivity among related channel targets. Positions 6, 9, 18, and possibly 20 may represent such modulatory sites in α-KTx15 toxins. These observations are based on a limited number of toxin-channel combinations and should therefore be regarded as a hypothesis for future comparative studies rather than as a general model of α-KTx evolution.

In conclusion, this study identifies BlTx2 as a highly informative α-KTx15 peptide for dissecting the molecular basis of Kv4.1-selective inhibition. We show that BlTx2 uses conserved α-KTx functional residues, including Lys27, Asn30, and Tyr36, supporting a canonical pore-blocking mechanism, but that its potency and selectivity are shaped by additional residues distributed across the toxin-channel interface. On the channel side, substitution of selected Kv4.1 pore-region residues with the corresponding residues present in Kv4.2 and Kv4.3 revealed that the chemical identity of positions 353, 355, and 381 strongly influences BlTx2 sensitivity. Most notably, the introduction of the Kv4.2/Kv4.3 lysine at position 355, replacing the Kv4.1 asparagine, abolished detectable inhibition. These results indicate that Kv4.1 selectivity is shaped not only by favorable toxin-channel contacts but also by the absence of residues that interfere with toxin binding or pore blockade. On the toxin side, mutational analysis separated residues required for Kv4.1 inhibition from residues that tune potency and cross-reactivity. In particular, Gln9 and Ile18 contribute to the restricted activity profile of BlTx2, as their replacement with lysine conferred Kv1.2 inhibition, an effect influenced by Kv1.2 Glu355. Together, these findings indicate that BlTx2 combines a conserved α-KTx pore-blocking core with peripheral residues that modulate potency and channel selectivity. This information may guide the rational engineering of Kv-channel ligands with tailored selectivity, either by strengthening desired interactions or by eliminating contacts that promote off-target activity. Because Kv4 channels contribute to transient A-type currents in neurons, the heart, and other excitable tissues, BlTx2 may serve as a useful research tool for probing Kv4.1 function in native systems, where broad-spectrum blockers or genetic manipulation can be difficult to interpret. Although medical applications would require extensive optimization of stability, delivery, safety, and immunogenicity, the selectivity of BlTx2 provides a potential starting point for developing Kv4.1-directed molecular probes or lead compounds for conditions in which Kv4.1-dependent excitability is altered.

## 4. Materials and Methods

### 4.1. Animals, Yeast, and Bacteria

*Escherichia coli* XL1 Blue cells [recA1 endA1 gyrA96 thi-1 hsdR17 supE44 relA1 lac F′ proAB lacIqZΔM15 Tn10(TetR)] were used for routine plasmid propagation. Recombinant toxins were expressed in *Saccharomyces cerevisiae* S-78 cells (Leu2, Ura3, Rep4). *Xenopus laevis* frogs were obtained from Xenopus 1, Inc. (Dexter, MI, USA). All procedures involving *Xenopus laevis* were approved by the Institutional Animal Care and Use Committee of Ben-Gurion University of the Negev and performed under protocol IL-15-04-2021(D).

### 4.2. Cloning, Expression, and Mutagenesis

Channel constructs were cloned into either pGEM or pRAT vectors. pRAT vectors contain a T7 RNA polymerase promoter for in vitro cRNA synthesis and *Xenopus laevis* β-actin 5′- and 3′-UTR sequences to support efficient expression in oocytes. Human Kv4.1, Kv4.1-KChIP2 chimera, Kv4.2, and Kv4.3 cDNA constructs were provided by Prof. Dirk Isbrandt (Bonn, Germany). A Kv4.1-Kv4.3 tandem dimer was constructed by adding a linker of 10 glutamine residues between the channels. Recombinant toxins were expressed from the yeast secretion vector pVT102U/α [58] provided by Prof. Chunguang Wang (Tongji University, China), as previously described for BlTx1 and BlTx2 [40]. This vector contains a URA3 selection marker, an α-mating factor secretion signal, and a KEX2 cleavage site upstream of the toxin coding sequence.

Yeast cultures were grown aerobically at 30 °C in SDD medium (0.67% yeast nitrogen base without amino acids, 0.14% yeast synthetic drop-out medium without histidine, leucine, tryptophan, and uracil, and 2% glucose). For a solid medium, 2% agar was added. Plasmid DNA was purified using the Wizard Plus SV Miniprep kit (Promega Corporation, Madison, WI, USA). Point mutations were generated by QuikChange site-directed mutagenesis (Stratagene, La Jolla, CA, USA) and verified by sequencing, or were synthesized by Twist Bioscience (South San Francisco, CA, USA). cRNA was transcribed in vitro using T7 RNA polymerase and the AmpliCap High Yield Message Maker kit (Epicentre, Madison, WI, USA). PCR reactions were performed using Phusion DNA polymerase (New England Biolabs, Ipswich, MA, USA) in a Labcycler 48 thermocycler (SensoQuest GmbH, Göttingen, Germany).

### 4.3. Electrophysiology

*Xenopus laevis* oocytes were isolated and injected with 20–40 nL of solution containing 0.3–40 ng cRNA using a Drummond manual oocyte microinjection pipette (3-000-510-X) with Drummond 3.5-inch glass capillaries (3-000-203-G/X) (Drummond Scientific Company, Broomall, PA, USA), pulled with a Sutter P-97 puller (Sutter Instrument Company, Novato, CA, USA). cRNA was injected in OR-2 solution containing, in mM: 82.5 NaCl, 2.5 KCl, 1 MgCl_2_, and 5 HEPES, pH 7.4. Injected oocytes were maintained in ND-91 solution containing, in mM: 91 NaCl, 2 KCl, 1.8 CaCl_2_, 1 MgCl_2_, and 5 HEPES, pH 7.4, supplemented with 100 U/mL penicillin, 100 μg/mL streptomycin, and 50 μg/mL gentamicin. Whole-cell currents were recorded 1–3 days after injection using the two-electrode voltage-clamp (TEVC) technique with an Axon Axoclamp 900A microelectrode amplifier (Molecular Devices, San Jose, CA, USA). Data were sampled at 5 kHz, filtered at 2 kHz, and acquired using Clampex 10.0 software (Molecular Devices). Recording electrodes were filled with 3 M KCl. The bath solution contained, in mM: 96 NaCl, 4 KCl, 1 MgCl_2_, 0.3 CaCl_2_, and 5 HEPES, adjusted to pH 7.4 with NaOH. TEVC recordings were performed using a repetitive voltage-step protocol. Oocytes were held at −80 mV, depolarized to +40 mV for 200 ms, stepped to −120 mV for 40 ms, and then returned to −80 mV. Voltage pulses were delivered every 5 s. Currents were recorded for 10 min before toxin application to establish a stable baseline. Toxins were applied for 10 min, unless otherwise indicated. Peak currents at +40 mV measured immediately before toxin application and after 10 min of toxin exposure were used to calculate the percentage of current block. The potency of BlTx2 on the Kv4.1-KChIP2 chimera was previously shown to be comparable to its potency on Kv4.1 alone [40]. Therefore, to elevate Kv4.1 expression levels, the activity of WT BlTx2 and all BlTx2 variants was tested using the Kv4.1-KChIP2 chimera, which is referred to hereafter as Kv4.1.

### 4.4. Isolation and Purification of Recombinant Toxins

Recombinant toxins were purified from the culture medium of yeast cells grown for three days in SDD minimal medium, using the purification workflow previously established for BlTx1 and BlTx2 [40]. Briefly, secreted proteins were concentrated by adsorption to SOURCE 15RPC resin (GE Healthcare Bio-Sciences AB, Uppsala, Sweden) after acidification of the culture medium with 0.1% (*v*/*v*) TFA. The resin was incubated with the medium for 15 min at room temperature with gentle agitation, collected by centrifugation at 2000× *g* for 6 min, and washed twice with 5% acetonitrile in 0.1% TFA. Bound proteins were eluted with 50% acetonitrile in 0.1% TFA, lyophilized, and resuspended in 20 mM ammonium acetate. Further purification was performed by reverse-phase HPLC on an ÄKTA basic purifier system (GE Healthcare Bio-Sciences AB). The initial separation was carried out on a 3 mL Resource RPC column using buffer A, 0.1% TFA in water, and buffer B, 0.1% TFA in acetonitrile, with the following gradient: 100% buffer A for 3 column volumes (CV), 0–10% buffer B for 1 CV, 10–40% buffer B for 15 CV, and 40–100% buffer B for 2 CV. Active fractions were subjected to final purification on a Vydac C18 column (4.6 × 250 mm; Grace, Hesperia, CA, USA) using 100% buffer A for 2.5 CV, 0–10% buffer B for 1 CV, 10–30% buffer B for 10 CV, and 30–100% buffer B for 2 CV. Toxin concentrations were determined from absorbance at 205 nm using sequence-specific ε205 values calculated as described previously [59]. Toxin variants with no detectable activity were purified as above without measuring activity, and MALDI-TOF was used to verify their identity. MALDI-TOF mass spectra were acquired at the Ilse Katz Institute for Nanoscale Science & Technology at Ben Gurion University using a MALDI-TOF/TOF Autoflex Speed mass spectrometer (Bruker Daltonics GmbH, Bremen, Germany), equipped with a smartbeam-II solid-state laser (modified Nd:YAG laser) λ = 355 nm.

### 4.5. 3D Modeling

Structural models were generated to guide the interpretation of toxin-channel interactions and to assist in the design of site-directed mutants. Homology models of BlTx1, BlTx2, and selected BlTx2-derived variants were generated using SWISS-MODEL [60], with α-KTx15.8 as the structural template for the toxin fold (PDB ID: 6AY8_A). To compare structural models of BlTx2 and selected BlTx2 variants, we used the AlphaFold Server (AlphaFold 3) [61] with mature toxin amino acid sequences as input. To model toxin binding to Kv1.2, the crystal structure of the Kv1.2-2.1 paddle chimera channel in complex with charybdotoxin (PDB ID: 4JTA) [15] was used as a reference toxin-channel complex. The channel coordinates were retained, and charybdotoxin was replaced by the corresponding modeled BlTx toxin through structural superposition, thereby preserving a canonical pore-blocking orientation similar to that observed for charybdotoxin.

To model BlTx2 binding to Kv4.1, a homology model of human Kv4.1 was generated using the cryo-EM structure of human Kv4.2 as the template (PDB ID: 7F0J) [62]. Four modeled Kv4.1 subunits were aligned to the pore-forming region of the Kv1.2-2.1 paddle chimera in the charybdotoxin-bound structure, and the modeled BlTx2 structure was positioned by superposition onto charybdotoxin. The resulting toxin-channel complexes were energy-minimized using Swiss-PdbViewer/DeepView version 4.1 [63], with 1000 steps of steepest descent, followed by 10,000 steps of conjugate gradient minimization and an additional 1000 steps of steepest descent. The minimized complexes were inspected to identify candidate toxin-channel residue pairs based on spatial proximity, steric compatibility, hydrophobic packing, and potential electrostatic complementarity. These models served as structural hypotheses for subsequent mutagenesis and functional interpretation.

To further refine the predicted toxin-channel complexes and sample alternative low-scoring binding conformations, the initial models were submitted to the ToxDock protocol through the Rosetta Online Server (ROSIE) [64,65]. The 25 top-ranked models, based on the reweighted complex score (reweighted_sc), were retained for further structural analysis. Interface properties, including the interface score (I_sc), buried interface surface area (I_bsa), and the number of predicted interfacial hydrogen bonds (I_hb), were considered alongside the overall ToxDock ranking to select representative models for subsequent structural analysis. Preservation of the canonical pore-blocking orientation and absence of major steric clashes were also considered. Both channel-toxin complex models are presented in the Supplementary Materials superimposed on the charybdotoxin-bound Kv1.2-2.1 paddle chimera structure (PDB ID: 4JTA; Figure S1). For selected point mutations used to assess local steric compatibility, mutant side chains were introduced using a rotamer library, and the rotamer with the most favorable local geometry and least apparent steric interference was selected.

### 4.6. Evolutionary Analysis

Nine additional transcripts corresponding to members of the α-KTx15 toxin family were identified in the core nucleotide database (core_nt) using BLASTN (BLAST+ version 2.17.0+, National Center for Biotechnology Information, Bethesda, MD, USA) [66] (AJ427743, KU253398, AJ427744, AJ850860, FJ360826, AF153692, KF612511, PX276998, and AF541980). Coding sequences were aligned using MUSCLE v3.8.31 [67]. Non-synonymous-to-synonymous substitution ratios (dN/dS) were estimated using the Selecton server version 2.2 [68] with models that allow site-specific variation in selective pressure, including MEC [68,69], M5, and M8 [70]. Model comparison showed a lower AIC score for MEC than for the null model M8a. Sites with dN/dS ≤ 0.2 across all three models were considered to be under strong purifying selection. Sites were classified as showing strong evidence for diversifying selection when dN/dS ≥ 2 in all three models. Sites not meeting this criterion, but showing dN/dS ≥ 1.5 in at least two models, were considered to show moderate evidence for diversifying selection. Sites that did not meet any of these criteria were not assigned to purifying or diversifying selection and were considered consistent with neutral evolution. The nucleotide sequence alignment and the corresponding Selecton outputs for the M5, M8, and MEC models are provided in Figure S5A–D.

### 4.7. Statistical Analysis

Data are presented as mean ± S.E.M., with n indicating the number of independent oocytes recorded for each condition. For each toxin concentration, measurements were obtained from 3 to 5 individual oocytes, with each oocyte exposed to only one toxin concentration and contributing a single observation. Concentration-response curves were fitted by nonlinear regression to all individual observations across concentrations; replicate measurements were not averaged before fitting, and individual oocytes were not fitted separately. Concentration-response relationships were fitted with a modified Hill equation: % inhibition = max/[1 + (IC_50_/[Tx])^h^], where max is the maximal inhibition, [Tx] is the toxin concentration, IC_50_ is the concentration producing 50% of maximal inhibition, and h is the Hill coefficient. Curve fitting and IC_50_ estimation were performed using GraphPad Prism 8 (GraphPad Software, Boston, MA, USA). Pairwise comparisons between concentration-response datasets were performed using an extra sum-of-squares F-test. Because each oocyte contributed only one observation, the measurements used in these curve comparisons were independent. *p* < 0.05 was considered statistically significant. For single-concentration inhibition experiments (BlTx2-I2E, Q3E, S5D, A14E, R19Q, V20E, and T21I), percentage current inhibition values obtained from four independent experiments per group were analyzed using one-way ANOVA followed by Dunnett’s multiple-comparison test, with WT as the control group. Statistical significance was defined as *p* < 0.05. Comparisons between WT and a single mutant channel were performed using a two-tailed Welch’s unpaired *t*-test. Reported *p* values are two-sided, and *p* < 0.05 was considered statistically significant.

## Figures and Tables

**Table 1:** Functional effects of BlTx1, BlTx2, and BlTx2 variants on Kv1.2 and Kv1.2-E355A. IC_50_ values and fold potency are shown for toxins tested on Kv1.2-WT and Kv1.2-E355A. IC_50_ values are presented as mean ± S.E.M. Fold potency was calculated as IC_50,reference_/IC_50,test_. For BlTx2-derived variants tested on Kv1.2-WT (a), fold potency was calculated relative to BlTx1 inhibition of Kv1.2-WT. For experiments with Kv1.2-E355A (b), fold potency was calculated relative to the activity of the same toxin variant on Kv1.2-WT. Thus, fold-potency values > 1 indicate increased apparent potency, whereas values <1 indicate decreased apparent potency. ND, IC_50_ could not be determined under the conditions tested.

Channel	Toxin	IC_50_ (nM)	Fold Potency	Figure
Kv1.2-WT	BlTx1	727 ± 86	---	Figure 3
Kv1.2-WT	BlTx2-WT	ND	---	Figure 3
Kv1.2-WT	BlTx2-Q9K	2492 ± 103	0.3 ± 0.04 ^a^	Figure 3
Kv1.2-WT	BlTx2-I18K	4488 ± 310	0.2 ± 0.02 ^a^	Figure 3
Kv1.2-WT	BlTx2-Q9K/I18K	596 ± 45	1.2 ± 0.2 ^a^	Figure 3
Kv1.2-E355A	BlTx2-Q9K	3748 ± 558	0.7 ± 0.1 ^b^	Figure 4
Kv1.2-E355A	BlTx2-I18K	9794 ± 691	0.5 ± 0.05 ^b^	Figure 4
Kv1.2-E355A	BlTx2-Q9K/I18K	1564 ± 100	0.4 ± 0.04 ^b^	Figure 4

**Table 2:** Functional effects of BlTx2 and Kv4.1 point mutations on inhibition. Single-amino-acid variants of BlTx2 were compared based on their inhibitory potency against WT Kv4.1, and WT BlTx2 was tested against the indicated Kv4.1 variants. IC_50_ values are presented as mean ± S.E.M. Fold potency was calculated as IC_50,reference_/IC_50,test_, using BlTx2-WT inhibition of Kv4.1-WT as the reference. Thus, fold-change values > 1 indicate increased apparent potency, whereas values <1 indicate decreased apparent potency. ND, IC_50_ could not be determined under the conditions tested. Statistical significance was determined relative to the inhibition of Kv4.1-WT by BlTx2-WT, as described in Section 4.7 * *p* < 0.05; ** *p* < 0.01.

Channel	Toxin	IC_50_ (nM)	Fold Potency	Figure
Kv4.1-WT	BlTx2 WT	35.6 ± 7.5	---	Figure 1, Figure 2, Figure 5 and Figure 6
Kv4.1-WT	N30A *	105 ± 20	0.3 ± 0.1	Figure 1
Kv4.1-WT	K27A	ND	---	Figure 1
Kv4.1-WT	Y36A	ND	---	Figure 1
Kv4.1/Kv4.3-chimera **	BlTx2 WT	1767 ± 203	0.02 ± 0.005	Figure 2
Kv4.1-T351S	BlTx2 WT	22.2 ± 4.5	1.6 ± 0.5	Figure 5
Kv4.1-N352S	BlTx2 WT	36.9 ± 13.2	1 ± 0.4	Figure 5
Kv4.1-K353A**	BlTx2 WT	1.8 ± 0.3	19.8 ± 5.3	Figure 5
Kv4.1-T354S	BlTx2 WT	18.8 ± 4.4	1.9 ± 0.6	Figure 5
Kv4.1-N355K	BlTx2 WT	ND	---	Figure 5
Kv4.1-S381K **	BlTx2 WT	350 ± 56	0.1 ± 0.03	Figure 5
Kv4.1-WT	I18M **	5.98 ± 0.89	5.9 ± 1.5	Figure 6
Kv4.1-WT	Q9Y **	8.01 ± 1.78	4.4 ± 1.4	Figure 6
Kv4.1-WT	I6K **	10.7 ± 2.1	3.3 ± 1	Figure 6
Kv4.1-WT	Q9D **	12.6 ± 1.3	2.8 ± 0.7	Figure 6
Kv4.1-WT	P37A	17.7 ± 2.7	2 ± 0.5	Figure 6
Kv4.1-WT	I6V	21.4 ± 3.3	1.7 ± 0.4	Figure 6
Kv4.1-WT	V34M	23.4 ± 4.9	1.5 ± 0.5	Figure 6
Kv4.1-WT	Q9A	24.6 ± 6.1	1.4 ± 0.5	Figure 6
Kv4.1-WT	I18D	29.4 ± 5.6	1.2 ± 0.3	Figure 6
Kv4.1-WT	A24V	35.9 ± 5.2	1 ± 0.2	Figure 6
Kv4.1-WT	Q1N	40.5 ± 8.9	0.9 ± 0.3	Figure 6
Kv4.1-WT	A25S	41.5 ± 11.7	0.9 ± 0.3	Figure 6
Kv4.1-WT	Q9N	50.0 ± 12.2	0.7 ± 0.2	Figure 6
Kv4.1-WT	A24N	51.3 ± 10.1	0.7 ± 0.2	Figure 6
Kv4.1-WT	R7E	65.1 ± 15.8	0.5 ± 0.2	Figure 6
Kv4.1-WT	R7D *	70.1 ± 15.3	0.5 ± 0.2	Figure 6
Kv4.1-WT	I29T *	82.3 ± 13.9	0.4 ± 0.1	Figure 6
Kv4.1-WT	A24S *	83.6 ± 19.7	0.4 ± 0.1	Figure 6
Kv4.1-WT	I18A *	97.2 ± 14.8	0.4 ± 0.1	Figure 6
Kv4.1-WT	S15A *	121 ± 19	0.3 ± 0.1	Figure 6
Kv4.1-WT	R7A **	173 ± 21	0.2 ± 0.05	Figure 6
Kv4.1-WT	S15D **	174 ± 41	0.2 ± 0.06	Figure 6
Kv4.1-WT	A25T **	260 ± 28	0.1 ± 0.03	Figure 6
Kv4.1-WT	A25N	ND	---	Figure 6

## Data Availability

The original contributions presented in this study are included in the article/Supplementary Material. Further inquiries can be directed to the corresponding author.

## Supplementary Materials

The following supporting information can be downloaded at: https://www.mdpi.com/article/10.3390/toxins18100431/s1, Figure S1: Structural comparison of the BlTx2-Kv1.2 and BlTx2-Kv4.1 models with the charybdotoxin-bound Kv1.2-2.1 paddle chimera structure; Figure S2: Biophysical properties of WT Kv1.2 and Kv1.2-E355A channels; Figure S3: Biophysical properties of WT Kv4.1 and mutant channels; Figure S4: AlphaFold models of BlTx2 and selected BlTx2 variants; Figure S5: Data used for evolutionary analysis of α-KTx15 family members.
