# Supplementary material for: The Architecture of Selectivity: Molecular Basis of BlTx2 Discrimination Among Kv Channels and Kv4 Isoforms
Source: Toxins (Basel). 2026 Oct 8;18(10):431. doi: 10.3390/toxins18100431 (PMC13709427; doi:10.3390/toxins18100431)
Supplement: Supplementary file 1 [file toxins-18-00431-s001.zip › toxins-4497708-supplementary.pdf]

Supplementary Materials: The Architecture of Selectivity: Molecular Basis of BlTx2 Discrimination Among Kv Channels and Kv4 Isoforms

Yuval Levy, Ayalon Reis, Galit Blecher, Oksana Daron, Sara Dadon, Raz Zarivach and Noam Zilberberg

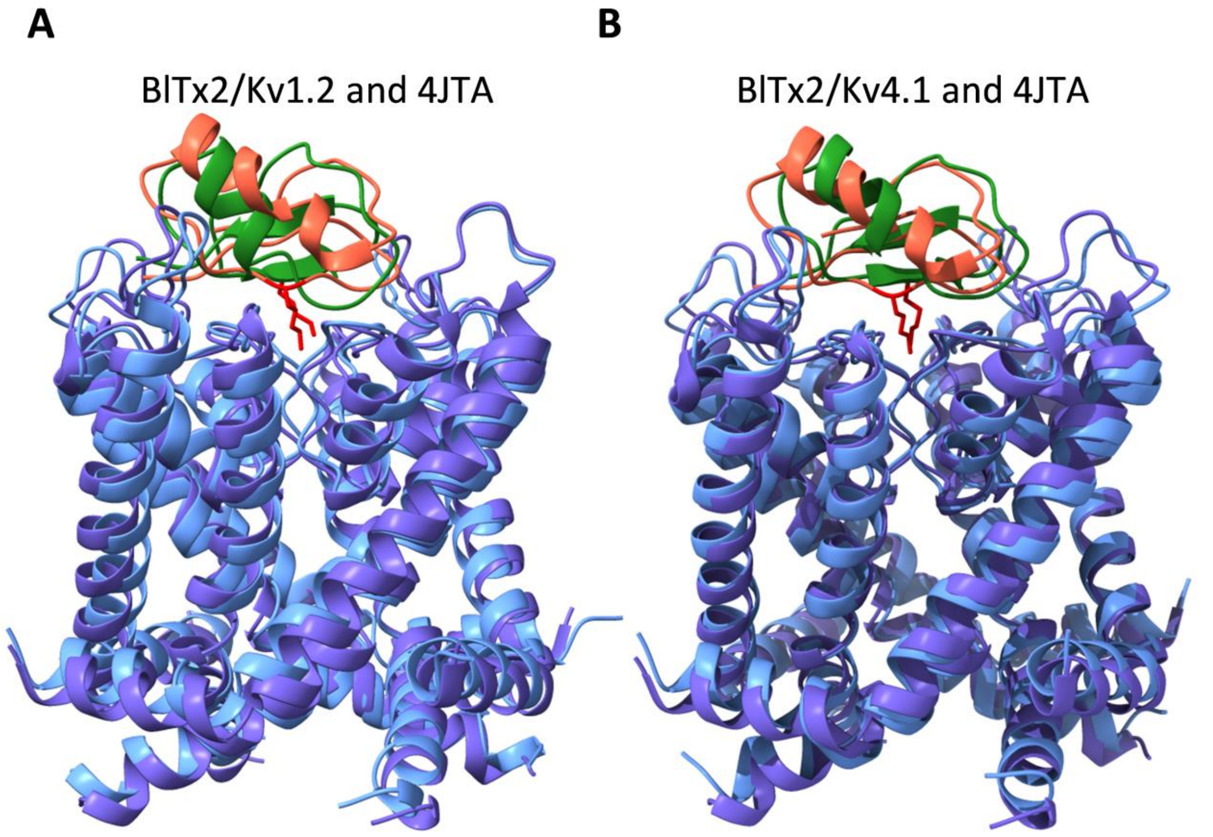

Figure S1. Structural comparison of the BlTx2-Kv1.2 (A) and BlTx2-Kv4.1 (B) models with the charybdotoxin-bound Kv1.2-2.1 paddle chimera structure (PDB ID: 4JTA). Channels and toxins are shown as ribbons. In the modeled complexes, the channels are shown in blue and BlTx2 in green. In the 4JTA reference structure, the Kv1.2-2.1 paddle chimera is shown in purple and charybdotoxin in red.

A

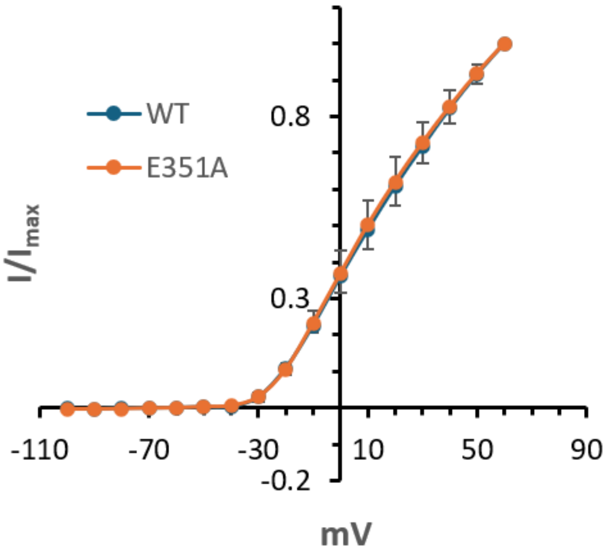

B

channel	n	$V_{1/2}$ (mV)	*P* vs WT	n	$\tau_{activation}$ (ms)	*P* vs WT
**WT-Kv1.2**	10	-4.06±1.61	-	10	2.59±0.30	-
**E355A**	10	-5.22±1.23	0.573	10	2.32±0.26	0.515

Figure S2. Biophysical properties of WT Kv1.2 and Kv1.2-E355A channels. A. Normalized current-voltage (I-V) relationships of WT Kv1.2 and Kv1.2-E355A channels. Each point represents the mean of 10 individual oocytes; error bars indicate SEM. For clarity, error bars are shown in the upward direction for WT Kv1.2 and in the downward direction for Kv1.2-E355A. B. Voltage dependence of activation and activation kinetics of WT Kv1.2 and Kv1.2-E355A channels. Conductance was calculated from the measured current according to ($G = I/(V-E_K)$), where E_K is the potassium equilibrium potential. For each individual oocyte, the normalized conductance-voltage relationship was fitted with a Boltzmann function, $G/G_{max} = 1/[1 + \exp((V_{1/2} - V)/k)]$, where $V_{1/2}$ is the voltage at half-maximal activation and k is the slope factor. $V_{1/2}$ values are presented as mean ± SEM; n indicates the number of individual oocytes analyzed. Activation time constants ($\tau_{activation}$) at +40 mV were obtained by fitting the rising phase of the current during the initial 10 ms with a single-exponential function using Clampfit 11. Reported *P* values were obtained using a two-tailed Welch's unpaired t-test. The mutation did not significantly alter either $V_{1/2}$ or the activation time constant.

A

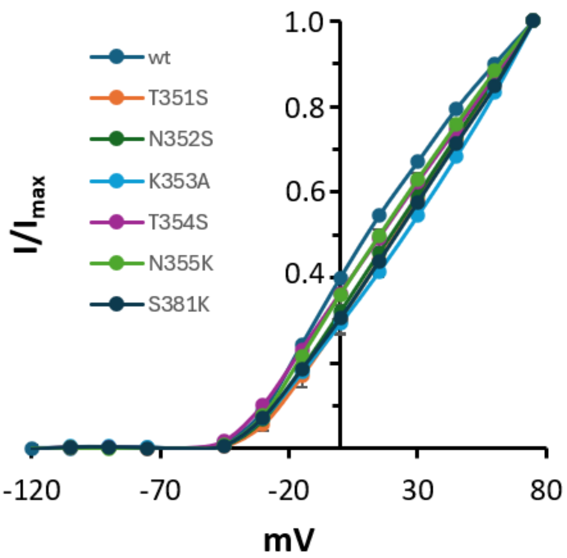

B

channel	n	$V_{1/2}$ (mV)	*P* vs WT	n	$\tau_{inactivation}$ (ms)	*P* vs WT
**WT-Kv4.1**	11	-12.71±1.43	-	13	49.4±2.8	-
**T351S**	16	-5.06±1.03	0.082	19	59.03±1.4	0.075
**N352S**	18	-6.21±1.9	0.102	18	58.1±1.9	0.133
**K353A**	16	-5.46±2.98	0.063	13	66.8±3.6	0.001
**T354S**	16	-12.93±1.86	1.000	16	65.8±3.8	0.001
**N355K**	10	-11.49±0.33	0.998	10	50.1±1.4	1.000
**S381K**	11	-5.85±2.06	0.132	10	61.3±5.0	0.054

Figure S3. Biophysical properties of WT Kv4.1 and mutant channels. A. Normalized current-voltage (I-V) relationships of WT Kv4.1 and the indicated mutant channels. Each point represents the mean of 11–18 individual oocytes; error bars indicate SEM and, where not visible, are smaller than the symbols. **B.** Voltage dependence of activation and inactivation kinetics of WT Kv4.1 and mutant channels. Condactance and $V_{1/2}$ values were calculated as in Figure S2. $V_{1/2}$ values are presented as mean ± SEM; n indicates the number of individual oocytes analyzed. Inactivation time constants ($\tau_{inactivation}$) at +60 mV were obtained by fitting the initial 150 ms of current decay with a single-exponential function using Clampfit 11. Statistical comparisons between WT and mutant channels were performed using ordinary one-way ANOVA followed by Dunnett's multiple-comparisons test, with WT as the control. Reported *P* values are two-sided Dunnett-adjusted *P* values. None of the mutations significantly altered $V_{1/2}$ of activation. K353A and T354S significantly increased $\tau_{inactivation}$ compared with WT (P = 0.001 for both comparisons); the remaining mutants did not differ significantly from WT.

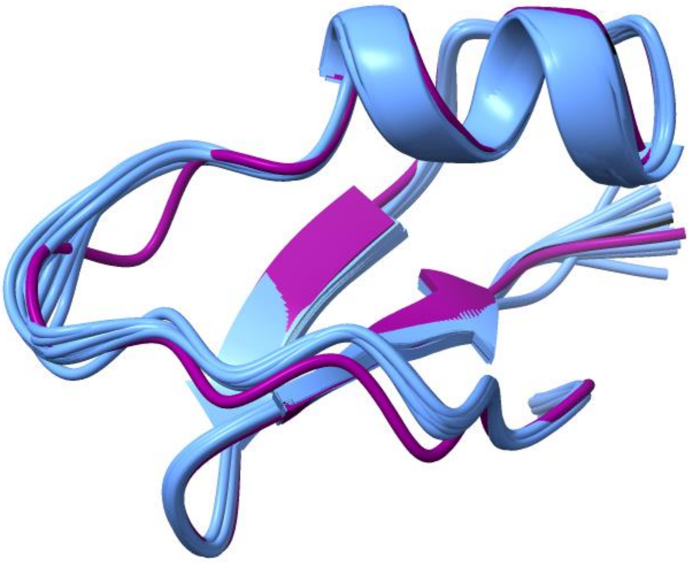

Figure S4. AlphaFold models of BlTx2 and selected BlTx2 variants. AlphaFold models were generated for wild-type BlTx2 and BlTx2 variants that exhibited significant changes in activity. The top-ranked model of each peptide is shown as a ribbon after Cα backbone alignment to the wild-type BlTx2 model. Wild-type BlTx2 is shown in black and is largely obscured by the superimposed variant models. BlTx2-I6K is shown in purple, and all other variants are shown in blue. Relative to wild-type BlTx2, the Cα RMSD values of all variants except BlTx2-I6K ranged from 0.606 to 0.847 Å, whereas BlTx2-I6K had a Cα RMSD of 1.065 Å. These low RMSD values support preservation of the predicted BlTx2 fold among the tested variants, although they do not substitute for experimental folding validation. Models shown are wild-type BlTx2, I6K, R7A, R7D, Q9Y, Q9D, S15A, S15D, I18A, I18M, A24S, A25N, A25T, K27A, N30A, and Y36A.

A

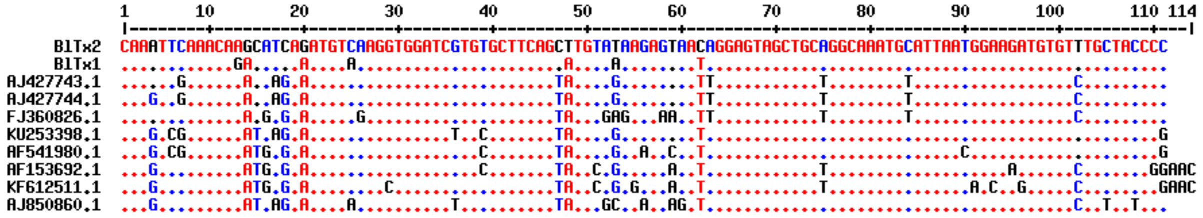

B

POS	AMINO	Ka/Ks	[Confidence Interval](* POSTERIOR PROBABILITIES		w =	0.0014	0.015	0.053	0.13	0.25	0.47
1	Q	0.16	[0.0014,0.87]	0.2	0.19	0.18	0.16	0.13	0.09	0.047	0.0057
2	I	0.97	[0.13,2.2]	5.50E-06	0.00058	0.0067	0.033	0.11	0.24	0.39	0.23
3	E	0.92	[0.13,2.2]	6.10E-06	0.00065	0.0074	0.037	0.11	0.25	0.39	0.2
4	T	0.1	[0.0014,0.47]	0.24	0.23	0.2	0.16	0.11	0.054	0.016	0.00036
5	N	0.74	[0.13,2.2]	9.50E-06	0.001	0.011	0.053	0.15	0.3	0.38	0.1
6	K	2.2	[2.2,2.2]*	5.60E-22	7.40E-15	4.70E-11	2.00E-08	2.20E-06	0.00013	0.0064	0.99
7	K	0.29	[0.015,0.87]	0.0044	0.044	0.13	0.23	0.28	0.22	0.086	0.0021
8	C	0.13	[0.0014,0.87]	0.21	0.21	0.19	0.16	0.12	0.075	0.031	0.0021
9	Q	1.5	[0.25,2.2]	6.20E-09	6.90E-06	0.00028	0.0034	0.022	0.096	0.31	0.57
10	G	0.38	[0.015,0.87]	0.003	0.03	0.096	0.18	0.26	0.26	0.16	0.016
11	G	0.089	[0.0014,0.47]	0.25	0.24	0.21	0.15	0.096	0.043	0.011	0.00024
12	S	0.31	[0.0014,2.2]	0.15	0.15	0.15	0.14	0.13	0.12	0.097	0.049
13	C	0.14	[0.0014,0.87]	0.21	0.2	0.19	0.16	0.12	0.081	0.037	0.0035
14	A	0.12	[0.0014,0.87]	0.22	0.21	0.19	0.16	0.12	0.071	0.028	0.0016
15	S	0.16	[0.0014,0.87]	0.2	0.19	0.18	0.16	0.13	0.091	0.047	0.0064
16	V	0.35	[0.015,0.87]	0.0034	0.034	0.11	0.2	0.27	0.25	0.13	0.0079
17	C	0.14	[0.0014,0.87]	0.21	0.2	0.19	0.16	0.12	0.078	0.034	0.0027
18	R	1.9	[0.87,2.2]	5.90E-17	7.30E-11	1.20E-07	1.80E-05	0.00074	0.014	0.16	0.83
19	R	0.77	[0.13,2.2]	5.80E-08	6.30E-05	0.0023	0.024	0.12	0.32	0.45	0.094
20	V	1.8	[0.47,2.2]	3.30E-14	3.90E-09	1.90E-06	0.00012	0.0026	0.03	0.21	0.75
21	I	0.36	[0.015,0.87]	0.0032	0.032	0.1	0.19	0.26	0.25	0.14	0.0095
22	G	0.089	[0.0014,0.47]	0.25	0.24	0.21	0.15	0.096	0.043	0.011	0.00024
23	V	0.097	[0.0014,0.47]	0.24	0.23	0.2	0.16	0.1	0.051	0.014	0.00028
24	A	0.12	[0.0014,0.87]	0.22	0.21	0.19	0.16	0.12	0.071	0.028	0.0016
25	A	0.11	[0.0014,0.47]	0.23	0.22	0.2	0.16	0.11	0.058	0.018	0.00057
26	G	0.16	[0.0014,0.87]	0.2	0.19	0.18	0.16	0.13	0.088	0.045	0.0056
27	K	0.083	[0.0014,0.47]	0.26	0.24	0.21	0.15	0.092	0.039	0.0081	7.70E-05
28	C	0.17	[0.0014,0.87]	0.19	0.19	0.18	0.16	0.13	0.094	0.052	0.008
29	I	0.13	[0.0014,0.87]	0.21	0.21	0.19	0.16	0.12	0.073	0.03	0.0018
30	N	0.1	[0.0014,0.47]	0.24	0.23	0.2	0.16	0.11	0.053	0.015	0.00033
31	G	0.43	[0.015,0.87]	0.0024	0.024	0.08	0.16	0.25	0.27	0.18	0.023
32	R	0.3	[0.015,0.87]	0.0043	0.043	0.13	0.23	0.28	0.22	0.093	0.0036
33	C	0.13	[0.0014,0.87]	0.21	0.21	0.19	0.16	0.12	0.075	0.031	0.0021
34	V	0.16	[0.0014,0.87]	0.2	0.19	0.18	0.16	0.13	0.09	0.047	0.0059
35	C	0.18	[0.0014,0.87]	0.19	0.18	0.18	0.16	0.13	0.097	0.055	0.0097
36	Y	0.19	[0.0014,0.87]	0.18	0.18	0.17	0.16	0.13	0.1	0.06	0.012
37	P	1	[0.053,2.2]	0.00084	0.0087	0.03	0.068	0.12	0.2	0.28	0.29
38	-	0.4	[0.0014,2.2]	0.14	0.14	0.14	0.13	0.13	0.12	0.11	0.085

Job Title: M5

C

POS	AMINO	Ka/Ks	[Confidence Interval](POSTERIOR PROBABILITIES									
					w =	0.0059	0.028	0.062	0.11	0.17	0.25	0.36
1	Q	0.15	[0.0059,0.59]	0.16	0.16	0.15	0.14	0.13	0.11	0.09	0.062	0.0026
2	I	0.82	[0.11,2.6]	0.00011	0.0024	0.011	0.031	0.067	0.12	0.21	0.36	0.19
3	Q	0.75	[0.11,2.6]	0.00012	0.0027	0.012	0.033	0.072	0.13	0.22	0.37	0.16
4	T	0.12	[0.0059,0.59]	0.2	0.19	0.17	0.14	0.12	0.091	0.063	0.031	0.0001
5	S	0.53	[0.11,2.6]	0.00017	0.0037	0.016	0.044	0.091	0.16	0.25	0.37	0.063
6	I	2.6	[2.6,2.6]*	4.60E-18	2.60E-13	6.70E-11	3.10E-09	6.40E-08	8.90E-07	1.10E-05	0.00024	1
7	R	0.24	[0.028,0.59]	0.012	0.055	0.1	0.15	0.18	0.2	0.18	0.12	0.00049
8	C	0.14	[0.0059,0.59]	0.18	0.17	0.16	0.14	0.12	0.1	0.079	0.048	0.00083
9	Q	1.9	[0.25,2.6]	5.60E-07	6.00E-05	0.00062	0.003	0.01	0.028	0.073	0.21	0.68
10	G	0.29	[0.028,0.59]	0.0093	0.042	0.083	0.13	0.17	0.19	0.2	0.17	0.0062
11	G	0.11	[0.0059,0.36]	0.21	0.2	0.17	0.14	0.11	0.084	0.054	0.024	8.20E-05
12	S	0.27	[0.0059,2.6]	0.13	0.13	0.13	0.13	0.12	0.12	0.11	0.097	0.037
13	C	0.14	[0.0059,0.59]	0.17	0.16	0.15	0.14	0.12	0.11	0.084	0.054	0.0015
14	A	0.13	[0.0059,0.59]	0.18	0.17	0.16	0.14	0.12	0.1	0.076	0.045	0.00059
15	S	0.16	[0.0059,0.59]	0.16	0.16	0.15	0.14	0.12	0.11	0.09	0.063	0.0031
16	A	0.27	[0.028,0.59]	0.01	0.045	0.09	0.13	0.17	0.19	0.2	0.16	0.0024
17	C	0.14	[0.0059,0.59]	0.18	0.17	0.16	0.14	0.12	0.1	0.082	0.051	0.0011
18	I	2.5	[0.59,2.6]	3.40E-13	3.90E-09	4.10E-07	9.60E-06	0.00011	0.00086	0.0056	0.046	0.95
19	R	0.56	[0.17,2.6]	4.70E-06	0.00047	0.0045	0.019	0.057	0.13	0.26	0.46	0.059
20	V	2.4	[0.59,2.6]	4.80E-11	1.10E-07	5.50E-06	7.60E-05	0.00058	0.0032	0.015	0.083	0.9
21	T	0.27	[0.028,0.59]	0.0097	0.044	0.087	0.13	0.17	0.19	0.2	0.16	0.003
22	G	0.11	[0.0059,0.36]	0.21	0.2	0.17	0.14	0.11	0.084	0.054	0.024	8.20E-05
23	V	0.11	[0.0059,0.59]	0.2	0.19	0.17	0.14	0.12	0.089	0.061	0.029	7.90E-05
24	A	0.13	[0.0059,0.59]	0.18	0.17	0.16	0.14	0.12	0.1	0.076	0.045	0.00059
25	A	0.12	[0.0059,0.59]	0.2	0.18	0.16	0.14	0.12	0.094	0.066	0.035	0.00019
26	G	0.15	[0.0059,0.59]	0.17	0.16	0.15	0.14	0.12	0.11	0.088	0.06	0.0027
27	K	0.1	[0.0059,0.36]	0.22	0.2	0.17	0.14	0.11	0.08	0.05	0.021	1.80E-05
28	C	0.16	[0.0059,0.59]	0.16	0.16	0.15	0.14	0.13	0.11	0.093	0.066	0.004
29	I	0.13	[0.0059,0.59]	0.18	0.17	0.16	0.14	0.12	0.1	0.078	0.047	0.00067
30	N	0.12	[0.0059,0.59]	0.2	0.19	0.17	0.14	0.12	0.091	0.063	0.031	9.40E-05
31	G	0.31	[0.028,0.59]	0.0076	0.035	0.073	0.12	0.16	0.19	0.21	0.2	0.0096
32	R	0.24	[0.028,0.59]	0.012	0.054	0.1	0.15	0.18	0.19	0.18	0.12	0.0011
33	C	0.14	[0.0059,0.59]	0.18	0.17	0.16	0.14	0.12	0.1	0.079	0.048	0.00083
34	V	0.15	[0.0059,0.59]	0.16	0.16	0.15	0.14	0.12	0.11	0.09	0.062	0.0028
35	C	0.17	[0.0059,0.59]	0.16	0.15	0.15	0.14	0.13	0.11	0.095	0.069	0.0051
36	Y	0.17	[0.0059,0.59]	0.16	0.15	0.14	0.14	0.13	0.11	0.097	0.072	0.0064
37	P	0.97	[0.062,2.6]	0.0034	0.016	0.035	0.059	0.088	0.12	0.17	0.23	0.28
38	-	0.35	[0.0059,2.6]	0.12	0.12	0.12	0.12	0.12	0.11	0.11	0.11	0.07

Job Title: M8

D

POS	AMINO	Ka/Ks	[Confidence Interval](* POSTERIOR PROBABILITIES									
				Ka/Ks =		6.60E-07	0.0001	0.0018	0.014	0.065	0.23	0.75
1	Q	0.11	[6.6e-07,3.3]	0.17	0.17	0.17	0.17	0.14	0.094	0.025	4.20E-05	0.059
2	I	0.96	[0.23,3.3]	1.80E-12	4.30E-08	1.40E-05	0.00078	0.015	0.14	0.52	0.092	0.22
3	Q	0.91	[0.23,3.3]	9.90E-13	2.30E-08	7.60E-06	0.00041	0.0081	0.069	0.2	0.0098	0.71
4	T	0.059	[6.6e-07,0.75]	0.19	0.19	0.19	0.18	0.15	0.081	0.013	4.60E-06	0.017
5	S	0.82	[0.23,3.3]	2.20E-12	5.10E-08	1.70E-05	0.00091	0.017	0.13	0.28	0.0048	0.56
6	I	3.3	[3.3,3.3]*	3.60E-28	2.00E-19	2.20E-14	7.00E-11	3.60E-08	8.50E-06	0.0022	1	0.00043
7	R	0.45	[0.014,3.3]	2.00E-06	0.0003	0.0055	0.04	0.16	0.37	0.3	0.0027	0.13
8	C	0.11	[6.6e-07,3.3]	0.17	0.17	0.17	0.16	0.15	0.11	0.042	0.00042	0.035
9	Q	1	[0.75,3.3]	7.70E-19	2.80E-12	1.60E-08	6.70E-06	0.00062	0.019	0.18	0.043	0.76
10	G	0.57	[0.014,3.3]	1.40E-06	0.00022	0.004	0.029	0.12	0.29	0.29	0.0072	0.26
11	G	0.091	[6.6e-07,0.75]	0.17	0.17	0.17	0.17	0.15	0.12	0.05	0.001	0.011
12	S	0.24	[6.6e-07,3.3]	0.14	0.14	0.14	0.14	0.13	0.1	0.056	0.0032	0.15
13	C	0.12	[6.6e-07,3.3]	0.16	0.16	0.16	0.16	0.15	0.11	0.047	0.00078	0.044
14	A	0.079	[6.6e-07,3.3]	0.18	0.18	0.18	0.17	0.15	0.084	0.016	1.00E-05	0.035
15	S	0.11	[6.6e-07,3.3]	0.18	0.18	0.18	0.17	0.14	0.072	0.01	2.90E-06	0.07
16	A	0.55	[0.014,3.3]	1.90E-06	0.00029	0.0052	0.038	0.15	0.29	0.17	0.00082	0.35
17	C	0.11	[6.6e-07,3.3]	0.17	0.17	0.17	0.16	0.15	0.11	0.045	0.00059	0.04
18	I	2.3	[0.75,3.3]	1.30E-25	7.10E-17	7.60E-12	2.40E-08	1.10E-05	0.0013	0.061	0.57	0.37
19	R	1.2	[0.23,3.3]	1.30E-18	4.80E-12	2.90E-08	1.20E-05	0.0011	0.034	0.33	0.14	0.49
20	V	1.2	[1,3.3]	3.50E-26	1.90E-17	2.10E-12	6.40E-09	2.80E-06	0.00034	0.013	0.088	0.9
21	T	0.62	[0.014,3.3]	1.50E-06	0.00023	0.0041	0.03	0.12	0.26	0.18	0.0014	0.41
22	G	0.091	[6.6e-07,0.75]	0.17	0.17	0.17	0.17	0.15	0.12	0.05	0.001	0.011
23	V	0.058	[6.6e-07,0.75]	0.19	0.19	0.19	0.18	0.15	0.083	0.015	1.60E-05	0.015
24	A	0.079	[6.6e-07,3.3]	0.18	0.18	0.18	0.17	0.15	0.084	0.016	1.00E-05	0.035
25	A	0.059	[6.6e-07,0.75]	0.19	0.19	0.19	0.18	0.15	0.074	0.01	3.10E-06	0.021
26	G	0.15	[6.6e-07,3.3]	0.16	0.16	0.16	0.15	0.14	0.11	0.056	0.002	0.058
27	K	0.061	[6.6e-07,0.75]	0.18	0.18	0.18	0.18	0.15	0.095	0.023	4.40E-05	0.0083
28	C	0.16	[6.6e-07,3.3]	0.15	0.15	0.15	0.15	0.14	0.11	0.059	0.0022	0.066
29	I	0.093	[6.6e-07,3.3]	0.17	0.17	0.17	0.17	0.15	0.097	0.027	0.0001	0.037
30	N	0.057	[6.6e-07,0.75]	0.19	0.19	0.19	0.18	0.15	0.079	0.012	2.40E-06	0.018
31	G	0.78	[0.065,3.3]	8.60E-07	0.00013	0.0024	0.018	0.078	0.23	0.38	0.062	0.23
32	R	0.49	[0.014,3.3]	1.80E-06	0.00027	0.0048	0.035	0.15	0.35	0.33	0.009	0.12
33	C	0.11	[6.6e-07,3.3]	0.17	0.17	0.17	0.16	0.15	0.11	0.042	0.00042	0.035
34	V	0.13	[6.6e-07,3.3]	0.16	0.16	0.16	0.16	0.14	0.11	0.041	0.0005	0.061
35	C	0.16	[6.6e-07,3.3]	0.15	0.15	0.15	0.15	0.14	0.11	0.061	0.0028	0.071
36	Y	0.19	[6.6e-07,3.3]	0.15	0.15	0.15	0.15	0.14	0.12	0.073	0.0068	0.078
37	P	1	[0.065,3.3]	4.20E-07	6.40E-05	0.0012	0.0086	0.039	0.12	0.24	0.1	0.49
38	-	0.42	[6.6e-07,3.3]	0.12	0.12	0.12	0.12	0.12	0.11	0.093	0.043	0.17

Job Title: MEC

Figure S5. Data used for evolutionary analysis of α-KTx15 family members. **A**. Alignment of the 10 nucleotide sequences used for the analysis. Only the regions encoding the mature toxins were included. Sequence alignment for the evolutionary analysis was performed using MUSCLE (Edgar, R.C. MUSCLE: Multiple sequence alignment with high accuracy and high throughput. Nucleic Acids Res. 2004, 32, 1792–1797), while MultAlin (Corpet F. Multiple sequence alignment with hierarchical clustering. Nucleic Acids Research. 1988;16(22):10881–10890) was used only to generate the visual representation shown in this panel. **B–D**. Selecton analysis using the M5 (B), M8 (C), and MEC (D) models. Colors indicate the inferred selective pressure at each residue, ranging from purifying selection (blue), through approximately neutral selection (white), to increasing evidence of positive selection (orange to red).
